# A Computational Analysis in a Cohort of Parkinson’s Disease Patients and Clock-Modified Colorectal Cancer Cells Reveals Common Expression Alterations in Clock-Regulated Genes

**DOI:** 10.3390/cancers13235978

**Published:** 2021-11-28

**Authors:** Müge Yalçin, Deeksha Malhan, Alireza Basti, Ana Rita Peralta, Joaquim J. Ferreira, Angela Relógio

**Affiliations:** 1 Institute for Theoretical Biology (ITB), Charité—Universitätsmedizin Berlin, Corporate Member of Freie Universität Berlin, Humboldt-Universität zu Berlin, 10117 Berlin, Germany; muege.yalcin@charite.de (M.Y.); deeksha.malhan@charite.de (D.M.); alireza.basti@charite.de (A.B.); 2 Molecular Cancer Research Center (MKFZ), Medical Department of Hematology, Oncology, and Tumour Immunology, Charité—Universitätsmedizin Berlin, Corporate Member of Freie Universität Berlin, Humboldt-Universität zu Berlin, 10117 Berlin, Germany; 3 Institute for Systems Medicine and Faculty of Human Medicine, MSH Medical School Hamburg, 20457 Hamburg, Germany; 4 EEG/Sleep Laboratory, Department Neurosciences and Mental Health, Hospital de Santa Maria—CHULN, 1649-035 Lisbon, Portugal; anaritaperalta@gmail.com; 5 Department of Neurology, Faculdade de Medicina, Universidade de Lisboa, 1649-028 Lisbon, Portugal; 6 Instituto de Fisiologia, Faculdade de Medicina, Universidade de Lisboa, 1649-028 Lisbon, Portugal; 7 CNS-Campus Neurológico Senior, 2560-280 Torres Vedras, Portugal; jferreira@medicina.ulisboa.pt; 8 Instituto de Medicina Molecular, Faculdade de Medicina, Universidade de Lisboa, 1649-028 Lisbon, Portugal; 9 Laboratory of Clinical Pharmacology and Therapeutics, Faculdade de Medicina, Universidade de Lisboa, 1649-028 Lisbon, Portugal

**Keywords:** circadian rhythms, colorectal cancer, Parkinson’s disease, high-throughput data, gene expression, bioinformatics, core-clock knockouts, survival analysis

## Abstract

**Simple Summary:**

Cancer and neurodegenerative diseases are two aging-related pathologies with differential developmental characteristics, but they share altered cellular pathways. Interestingly, dysregulations in the biological clock are reported in both diseases, though the extent and potential consequences of such disruption have not been fully elucidated. In this study, we aimed at characterizing global changes on common cellular pathways associated with Parkinson’s disease (PD) and colorectal cancer (CRC). We used gene expression data retrieved from an idiopathic PD (IPD) patient cohort and from CRC cells with unmodified versus genetically altered clocks. Our results highlight common differentially expressed genes between IPD patients and cells with disrupted clocks, suggesting a role for the circadian clock in the regulation of pathways altered in both pathologies. Interestingly, several of these genes are related to cancer hallmarks and may have an impact on the overall survival of colon cancer patients, as suggested by our analysis.

**Abstract:**

Increasing evidence suggests a role for circadian dysregulation in prompting disease-related phenotypes in mammals. Cancer and neurodegenerative disorders are two aging related diseases reported to be associated with circadian disruption. In this study, we investigated a possible effect of circadian disruption in Parkinson’s disease (PD) and colorectal cancer (CRC). We used high-throughput data sets retrieved from whole blood of idiopathic PD (IPD) patients and time course data sets derived from an in vitro model of CRC including the wildtype and three core-clock knockout (KO) cell lines. Several gene expression alterations in IPD patients resembled the expression profiles in the core-clock KO cells. These include expression changes in *DBP*, *GBA*, *TEF*, *SNCA*, *SERPINA1* and *TGFB1*. Notably, our results pointed to alterations in the core-clock network in IPD patients when compared to healthy controls and revealed variations in the expression profile of PD-associated genes (e.g., *HRAS* and *GBA*) upon disruption of the core-clock genes. Our study characterizes changes at the transcriptomic level following circadian clock disruption on common cellular pathways associated with cancer and neurodegeneration (e.g., immune system, energy metabolism and RNA processing), and it points to a significant influence on the overall survival of colon cancer patients for several genes resulting from our analysis (e.g., *TUBB6*, *PAK6*, *SLC11A1*).

## 1. Introduction

Several pathologies develop with aging. Among those, cancer and neurodegenerative disorders affect (in total) about 41.3 million people worldwide and are a major health problem [1,2]. These two disease groups seem to relate to opposite cellular fate phenotypes, which contribute to the pathological development. While cancer is linked to increased cellular proliferation and resistance to apoptosis, neurodegeneration involves the progressive death of neuronal cells [3]. In particular, tumorigenesis is characterized by the disruption of key molecular events, defined as the hallmarks of cancer [4]. These include aberrant cellular proliferation, resistance to cell death, induction of angiogenesis and metastasis, altered cellular energetics and evading immune system response. Neurodegeneration, on the other hand, is characterized by the excessive loss and malfunctioning of neurons, induced cell death and proteopathies such as accumulation of misfolded proteins [5]. The placement of neurodegeneration and cancer at seemingly opposite ends of specific disease mechanisms is further evidenced by the inverse correlation between cancer incidence and neurodegenerative disorders like PD that has been reported in a number of epidemiological studies [6,7,8]. However, more recently, it has been reported that PD patients are at greater risk of certain types of neoplasms, particularly of melanoma and brain cancer [9], suggesting putatively shared pathological mechanisms. In both diseases, the pathological development process requires a multistep accumulation of events that lead to irreversible cellular changes, as it is expressed in a linear relationship between age and increased incidence rate [10]. The hallmarks of physiologic aging and neurodegeneration include genomic instability, telomere attrition, epigenetic alterations, loss of proteostasis, deregulated nutrient sensing, mitochondrial dysfunction, cellular senescence, stem cell exhaustion, and altered intercellular communication [11,12]. Many of these processes are also relevant to tumorigenesis [3,6,13,14]. Genetic forms of PD are particularly interesting in this overlap. Parkin, an E3-ubiquitin ligase that regulates protein metabolism, mitochondrial function, mitophagy and redox homeostasis, responsible for one of the most common familial forms of PD and also implicated in IPD (PD of unknown genetic origin), is mutated in many somatic cancers, where it seems to act as a tumour suppressor gene [15]. Other genes implicated in genetic forms of Parkinsonism, like *PINK1* (PTEN-induced kinase 1), *LRRK2* (Leucine-rich repeat kinase 2), *PARK7* (Parkinsonism associated deglycase, also known as DJ-1) have also been shown to be relevant in the development of neoplasms, where they interfere in the regulation of processes such as cell cycle, apoptosis, mitochondrial homeostasis and oxidative stress. *SNCA* which encodes for α-synuclein in humans is one of the main proteins linked to Lewy bodies, which constitute the neuropathological hallmark of PD. Interestingly, α-synuclein is overexpressed in melanoma and other cancers. On the other hand, genes primarily implicated in cancer-related pathways, such as *TP53*, *MTOR*, *TSC1/2*, *PIN1*, *MC1R* and the *PTEN* pathways, have also been shown to be implicated in PD pathogenesis [16]. Thus, increasing evidence points to the disruption of common cellular events in both cancer and PD [2,3,4,5], but the mechanisms underlying these associations require further studies. These mechanisms include the regulation of cell cycle, DNA repair, immune response and redox homeostasis [3,6,13,14]. Recent work from our group using time series data for an in vitro model of colorectal cancer (CRC) progression pointed to a link between cancer and neurodegenerative diseases, involving the circadian system [17], and suggested that altered oscillatory patterns of cancer-associated genes may also be involved in neurodegeneration in Huntington’s disease, but also revealed differential enrichment of genes involved in Alzheimer’s disease and PD.

PD is the second most common neurodegenerative disorder, and it is expected to affect over 12 million people in the next 20 years [18]. Clinical features of PD include both motor (e.g., muscular rigidity) and nonmotor (e.g., fatigue and musculoskeletal pain) symptoms. There is currently no cure nor preventive treatment for PD. Despite the existence of multiple treatment strategies, motor disability progresses over time and nonmotor symptoms are frequently difficult to control [19]. A PD patient is also at increased risk of developing other clinical complications, which include sleep disorders [20], neuropsychiatric disorders [21], skin and brain cancer [9]. The underlying mechanisms leading to these associations are far from being understood.

Work from our group and others pointed to the involvement of the circadian system in the regulation of neurodegenerative pathways in the context of cancer cellular models [17,22], reviewed in [23]. The circadian clock is an internal timekeeping molecular machinery that generates oscillations in the expression of genes and proteins with a period of approximately 24 h [24,25]. Its proper functioning ensures appropriate timing of physiological processes such as regulation of hormonal activity, core body temperature, sleep/wake cycles and metabolism. At the molecular level, the circadian clock consists of transcriptional and translation feedback loops (TTFLs) involving several families of genes and proteins (CLOCK, BMAL, RORs, PERs, CRYs, REV-ERBs) [25,26]. These elements form the core-clock and drive 24-h rhythmic oscillations in the expression of various target genes known as clock-controlled genes (CCGs). CCGs are involved in the regulation of numerous cellular processes including DNA damage response [27], cell cycle [28,29,30], immune system [31], cell fate decisions [32] and metabolism [33]. The disruption of proper functioning of the circadian clock was found to be associated with several diseases including cancer [34,35], cardiovascular diseases [36], sleep disorders [37], and neurodegeneration [38].

Circadian misalignment in PD has been reported, both clinically and through animal models [39,40,41,42]. It can contribute to the appearance of disabling symptoms, both motor and nonmotor, but it can also play a crucial role in the physio-pathological mechanisms that lead to progressive neurodegeneration, providing novel symptomatic or even preventive therapeutic approaches. A recent study suggested that circadian disturbances, measured through actigraphy, antedate and predispose the development of PD during longitudinal follow-up in older men who had not been previously diagnosed with PD [43]. Whether these early changes constitute true risk factors or the manifestations of PD in its preclinical form [44] remains to be elucidated. Evidence of circadian dysfunction in PD was found also at the molecular level. Single nucleotide polymorphisms of several clock-related genes have been shown to be associated with particular PD phenotypes, like tremor-dominant (ARNTL rs900147 variant), postural instability/gait difficulty (PER1 rs2253820 variant) [45], presence of motor fluctuations, sleep disorders (CLOCK 3111T/C variant) [46] and depression (Tef rs738499 variant) [47].

The circadian expression profile of clock genes has been seldomly evaluated in PD. In a cohort of 17 PD patients *BMAL1*, *BMAL2*, *DEC1*, *CLOCK* and *PER1* expression were evaluated during the night, at four time points, from 21 h to 9 h, and compared between PD patients and a healthy control group (sex and age matched) [48,49]. The data showed a significant decrease in *BMAL1* and *BMAL2* expression at 21 h and 24 h (midnight) in PD patients compared to a control group of healthy subjects for the same time point. However, all other clock genes evaluated (*CLOCK*, *DEC1* and *PER1*) showed no significant differences. Breen and colleagues [50] quantified the expression of *BMAL1*, *PER2* and *REV-ERBα* in 30 newly diagnosed PD patients, during the day with three-hour intervals. The authors reported a lack of diurnal variation in *BMAL1* expression in PD. Furthermore, Pacelli and colleagues quantified the expression of various clock genes (*CLOCK*, *CRY1*, *CRY2*, *NR1D1*, *PER1*, *PER2*, *PER3*, *BMAL1*) in cultured fibroblasts from two PD patients with *PRKN* (Parkin) mutations and found an absence of circadian rhythmicity (period close to 24 h) particularly in *CLOCK*, *PER1* and *PER2* in fibroblasts from PD patients [22].

The mechanisms that underlie the relations between PD and the circadian clock are unknown, but they may include reciprocal regulations between clock genes and dopamine, interactions with the orexinergic system, oxidative stress and mitochondrial function and inflammation [51,52]. Visual dysfunction, extensively demonstrated in PD, may also play a role in circadian changes, given the fundamental role of light as an entrainment stimulus [53].

To further investigate the underlying role of the circadian clock in PD and cancer, we analysed microarray data sets obtained from a cohort of 205 IPD patients and 233 healthy controls [54]. We used colorectal cancer cell lines of different origins with distinct clock phenotypes to analyse the effects of clock disruption on cancer pathways, potentially related to PD. For this, we chose an in vitro CRC model with cells depicting a robust circadian clock (HCT116 and SW480 cell lines) and cells with a dysregulated clock (SW620, the metastatic counterpart of SW480 from the same patient, and HCT116 clock-KO cells). This allowed us also to investigate whether cancer cells of different progression stages correlate with pathways associated with clock disruption and PD.

Furthermore, and to attain a more mechanistic insight in the possible impact of circadian clock disruption in disease-associated pathways, including pathways related to cancer and PD pathogenesis, we complemented our analysis with newly generated time course RNA sequencing (RNA-seq) data sets for CRC cells including the wild type cells (HCT116^WT^) and three core-clock knockout (KO) cell lines (*ARNTL*^KO^, *PER2*^KO^ and *NR1D1*^KO^*)*. Our results show that core-clock disruptions affect average and rhythmic expression of genes involved in both cancer and neurodegeneration-related pathways. We found *HRAS* and *GBA* to be differentially rhythmic in *PER2*^KO^ cells as compared to the HCT116^WT^ cells, which are involved in cell proliferation and increased PD susceptibility, respectively [55]. In addition, we detected an overlap between genes differentially expressed upon core-clock KO in CRC and in IPD patients. These include *SERPINA1,* involved in protein metabolism and immune system, as well as *TGFB1*, a key component of TGF-β signalling pathway, which regulates cell proliferation, motility and differentiation. Furthermore, our analysis using a colon adenocarcinoma (COAD) data set retrieved from the TCGA (the cancer genome atlas) data base to assess the impact of these genes on cancer patient survival revealed four of these genes, namely *TUBB6*, *PAK6*, *SULT1A1*, *SLC11A1*, which are involved in cancer hallmarks [56], to have a significant impact on overall survival. Thus, our data reinforce the hypothesis of a common regulation of specific pathways and highlight a role for the circadian system and its disruption in cancer and PD.

New insights regarding the circadian rhythms of PD patients may thus contribute to a better understanding of the molecular basis of PD. Characterizing circadian oscillations in PD and cancer patients may provide a potential benefit for the diagnosis and monitoring of these two aging-related pathologies and can be further translated for treatment optimization.

## 2. Materials and Methods

### 2.1. Cell Culture

Human colorectal carcinoma cell line HCT116 (ATCC^®^ CCL-247™) and their derived knockout mutants lacking *ARNTL* (also known as *BMAL1*), *PER2* or *NR1D1* (also known as *REV-ERB α*), respectively (see Section 2.2) were cultured in Dulbecco’s Modified Eagle Medium (DMEM) (Gibco, Thermo Fisher Scientific, Waltham, MA, USA) supplemented with 10% FBS (Fetal Bovine Serum) (Gibco, Thermo Fisher Scientific, Waltham, MA, USA) and 1% Penicillin−Streptomycin (Gibco, Thermo Fisher Scientific, Waltham, MA, USA) in a humidified atmosphere containing 5% CO_2_ at 37 °C. A LUNA™ Automated Cell Counter (Logos Biosystems, Anyang, South Korea) was used for cell counting and morphology analysis. All cell lines were tested for mycoplasma contamination (Mycoplasmacheck, Eurofins Genomics, Ebersberg, Germany).

### 2.2. CRISPR-Cas9 Knockout Generation

HCT116 core-clock knock out mutants were generated using the CRISPR-Cas9 methodology. In short, WT cells were transfected with CRISPR-Cas9 plasmids containing GFP marker and guide RNAs targeting multiple exons of *ARNTL*, *PER2* or *NR1D1* genes. Cell transfection was performed using FuGENE HD Transfection Reagent (Promega, Madison, WI, USA) according to manufacturer’s instructions. Forty-eight hours after transfection, CRISPR/Cas9 GFP-positive cells were single-cell sorted into 96-well plates using an S3e cell sorter (Bio-Rad laboratories, Hercules, CA, USA). Cells were expanded and successful knockout clones were verified. For each knockout condition, several single clones were investigated on the DNA and RNA levels to characterize and confirm the knockout. Additionally, Sanger sequencing flanking the guide-RNA binding site was performed using the selected knockout clones in order to determine the genomic modification upon CRISPR-Cas9 editing. The KOs were further validated on the generated RNA-seq data using differential expression analysis on the ECCN (extended core-clock network) genes (Figure S1).

### 2.3. Sample Preparation for 45-h Time Course RNA-seq

HCT116 cells were seeded in triplicates (12-well plates, 2 × 10^5^ cells/well). Sampling was carried out from 9 to 54 h after synchronization (via medium change) in 3-h intervals. Synchronizing time cues (e.g., light) that enable the resetting and coordination of circadian rhythms are termed *Zeitgeber* and the time after entrainment to a given *Zeitgeber* is referred as ZT (*Zeitgeber* Time) [57,58]. In our study, synchronization of CRC cells was performed via medium exchange (as *Zeitgeber*), which induces the activity of elements of the clock machinery and subsequently the clock-controlled genes. Of note, the time post-synchronization in cultured cancer cell lines can be compared, to some extent, to time after light exposure (related to time of the day) in humans, as light serves as the main *Zeitgeber* in mammals. To synchronize the circadian clock in cell culture environment, different synchronization agents can be used (e.g., dexamethasone, forskolin and serum shock) [28,59,60,61]. We have previously shown that medium exchange is as effective as other mentioned agents in synchronizing the circadian clock in different colorectal cancer cell lines as measured by the *BMAL1*-promoter activity over time [62]. Based on these results, we used a simple medium exchange to synchronize the cells. Total RNA was isolated using the RNeasy Plus Mini kit (Qiagen, Hilden, Germany) according to the manufacturer’s instructions. A Nanodrop 1000 (Thermo Fisher Scientific, Waltham, MA, USA) was used to measure RNA concentration. RNA was stored at −80 °C until further usage mRNA libraries were prepared using the TruSeq Stranded mRNA Sample Preparation Kit (Illumina, San Diego, CA, USA) according to guidelines and sequenced at the European Molecular Biology Laboratory (EMBL) GeneCore Facility (EMBL, Heidelberg, Germany) on an Illumina NextSeq 500 platform (average depth: 100 M; 75-bp paired-end reads).

### 2.4. RNA-seq and Microarray Data Pre-Processing

Quality control of raw reads was carried out for HCT116 cells (Accession number: E-MTAB-9701) and SW480 (ATCC^®^ CCL-228™), SW620 (ATCC^®^ CCL-227™) (Accession number: E-MTAB-7779 [63]) cells RNA-seq data (75 bp paired-end) using FastQC (v0.11.7) [64]. Trimmotatic (v0.38, [65]) with TruSeq3-PE-2 was used to remove adapter sequences. STAR (v2.6.0a, [66]) algorithm was used to align the reads to the human genome (Homo_sapiens.GRCh38, Ensembl release 92). Translation to transcript coordinates was carried out with quantMode TranscriptomeSAM. and further quantification was performed using Salmon (v0.10.2, [67]) with default parameters and using the seqBias option. Transcripts per million (TPM) counts were scaled using the *tximport* package (v1.6.0, [68]) by first multiplying TPM with feature length and then scaling up to the library size (lengthScaledTPM), resulting in summarized gene-level (txOut = FALSE, based on Ensembl Transcript IDs) count estimates. Log2-transformation was performed using the cpm function and applying the TMM method via the R package edgeR (v3.20.9, [69]). Only genes with ≥0.5 CPM (average over all time points) were retained and counts were renormalized. Raw microarray dataset (GEO Accession number: GSE99039; date of data retrieval: 16.02.2021) consisting of data sets from 205 patients with IPD and 233 controls was downloaded using R package GEOquery [70]. Expression levels were calculated using the Robust Multi-Array Average (RMA) preprocessing procedure as implemented in R affy package [71]. The R package arrayQualityMetrics [72] was used for quality control and statistical testing of the arrays. After quality control (QC) to identify possible outliers, 6 arrays failed 2 or more QC tests and were removed. Following the removal of outliers and of 1 control participant with *PINK1* mutation, 204 IPD and 227 controls were retained for further analysis (Table S1). The data was then batch corrected using ComBat [73] and annotated using the hgu133plus2.db package (version 3.2.3, [74]). For genes that mapped to multiple probes, the probe with the highest mean expression was retained. A low expression filter was set based on the histogram of intensity distribution of genes. Genes that did not pass this expression cutoff (<4) in at least 204 samples (the smaller group of samples in the cohort) were removed.

### 2.5. Rhythmicity Analysis

Circadian and ultradian rhythms were detected with the RAIN algorithm [75]. Circadian-related parameters (phase and amplitude) were determined using the harmonic regression method as implemented in the R package HarmonicRegression (v1.91) [76], and setting the period to 24 h for circadian and 12 h or 8 h for ultradian rhythms. For the RNA-seq data sets (E-MTAB-9701 and E-MTAB-7779), unlogged (not log2-scaled) expression values were used as input. RAIN *p*-values were Benjamini-Hochberg (BH) adjusted for multiple testing. Statistical significance for 24 h rhythmic genes was set at *q* < 0.05 and a relative amplitude ≥ 0.1. Amplitudes reflecting the distance between maximum and minimum data values were estimated using harmonic regression based on the formula A= √ (a2 + b2) and phases reflecting the peak expression time formulated as tan φ = b/a. The DODR (Detection of Differential Rhythmicity) [77] method was used for differential rhythmicity analysis, and pairwise comparisons between HCT116^WT^ and each KO cell line were carried out. DODR *p*-values were BH adjusted for multiple testing and a threshold set for differentially rhythmic genes as *q* < 0.05.

### 2.6. Differential Expression Analysis

For the PD microarray datasets, differential expression analysis was performed using the R limma package [78]. A nominal *p* < 0.01 and a |log2 fold change (FC)| ≥ 0.1 was set for statistical significance of up- and down-regulation in gene expression. For RNA-seq datasets, a linear model including circadian harmonics was fitted on TMM normalized and mean-variance assessed (with voom) data. Subsequently, the coefficient representing the mean gene expression was used for comparisons of each knockout to the HCT116^WT^ condition using the R limma package. Statistical significance was set to *q* < 0.05 and |log2 FC| > 0.58 (corresponding to a |FC| > 1.5) for comparisons using only RNA-seq data, whereas a cutoff of *p* < 0.01 and an absolute log_2_ FC ≥ 0.1 was selected for comparison using both RNA-seq and microarray data sets. For the visualization of heatmaps, gene expression values were standardized using z-score transformation formulated as follows$:\;z-\mathrm{score}=\frac{\mathrm{expression}\;\mathrm{value}\;\left(x\right)-\;\mu \;\left(\mathrm{mean}\right)}{\sigma \left(\mathrm{standard}\;\mathrm{deviation}\right)}$.

### 2.7. Curation of KEGG Pathways

KEGG pathways known to be disrupted in cancer and neurodegeneration were pre-selected for the subsequent analysis. These included the “immune system” module, all pathways from “replication and repair” under “genetic information and processing” module and pathways from “cell growth and death” categorized under the “cellular processes” module [79,80]. Because there were no explicit redox homeostasis pathways in KEGG, we included human “energy metabolism” pathways and “transport and catabolism” pathways classified under “cellular processes”. In addition, we included the PD pathway (hsa05012) from the KEGG human disease modules. The KEGG PD pathway includes elements from several other KEGG pathways (calcium signalling; unfolded protein response; mitophagy; apoptosis; dopamine metabolism; oxidative phosphorylation; microtubule-based transportation and transcription). For curation of lists, we used KEGGREST (v1.30.1, [81]) as implemented in R package. Following the curation of the results, a unique list of genes was generated comprising 2507 unique genes.

### 2.8. Functional Enrichment

A functional enrichment analysis was performed with GO terms (biological process—BP) for significantly rhythmic genes in CRC cells and for differentially expressed genes in CRC cells and IPD datasets. The enrichment was performed using the clusterProfiler R package [82]. The corresponding results are provided in Tables S8–S10.

### 2.9. Clinical Characteristics of the Cohort of IPD Patients

According to the original study of Shamir and colleagues [54], all IPD patients were subjected to an extensive clinical evaluation in a tertiary referral central and met the United Kingdom Parkinson’s Disease Society Brain Bank Criteria except that positive family history was not set as an exclusion criterion. Patients with single-photon emission computer tomography (SPECT) data available were excluded from the IPD cohort if the dopaminergic deficits were not evident in the dopamine transporters scan (DaTSCAN). The raw microarray data sets (GEO Accession number: GSE99039, date of data retrieval: 16.02.2021) were retrieved from the Gene Expression Omnibus (GEO) repository. Demographic information on age and sex for controls and IPD patients, as well as age at disease onset, Hoehn and Yahr stages, and MoCA scores (Montreal cognitive assessment), are indicated for the complete dataset (GSE99039) in Table 1. Hoehn and Yahr (H and Y) scale and MoCA scores are indicated in Table 1. H and Y scale reflects the overall progression of disabilities and symptoms in PD, with higher scores indicating higher disability (ranging from 1: unilateral involvement only with minimal or no functional disability; to 5: confinement to bed or wheelchair unless aided) [83]. The MoCA test is a rapid screening instrument for mild cognitive dysfunction that assesses different cognitive domains. The total possible score is 30 points, and a score of 26 or above is considered normal.

### 2.10. TCGA Colon Adenocarcinoma (TCGA-COAD) Analysis

Colon adenocarcinoma RNA-Seq data sets (COAD, HiSeq 2000) and corresponding clinical information were retrieved from TCGA (available at https://portal.gdc.cancer.gov, Accessed date: 8 August 2021) using the TCGAbiolinks package [84,85]. Primary tumour (N = 285) and normal tissue samples (N = 41) were included in the analysis. Gene expression quantification files aligned to Human Genome (Homo sapiens, GRCh37, hg19) were retrieved for further processing from the legacy archive. Raw counts were normalized using TMM from edgeR package [69] and voom functionality from the limma package [78] used for assessment of the mean variance relationship of log-counts. A low gene expression filtering (logCPM > 0.5, in more than 50% of the total population) was used to define the gene expression cutoff. Following batch correction, assessed based on the plate information, differential expression analysis was performed using the limma package [78]. The *p*-values were BH adjusted for multiple testing and a cutoff of log FC > 1 and FDR *q* < 0.01 was used to determine the significance of differential expression (DE) by comparing the cancer tissue versus samples derived from the normal adjacent tissue. The resulting 3234 DE genes were subsequently analysed using Kaplan–Meier (KM) survival analysis to compute survival univariate curves. Out of the DE gene list, 289 were significantly associated with patient overall survival (*q* < 0.05). The clinical features regarding the last follow-up and days until death were used to assess the survival analysis for cancer tissue samples. Briefly, each gene expression value in cancer samples was divided into two groups based on the median expression level (high versus low expression) in all samples. KM survival analysis was performed between high and low expression groups using the ‘survival’ package (version 3.2-12). The fitted survival curves were visualized using survminer package (version 0.4.9). We additionally investigated the possible influence of clinical traits (sex, age, cancer stage) using the TCGA COAD datasets. Because stage correlated significantly with the survival data (Figure S15), we used a Cox Regression model by including stage as a stratification factor (the list of survival associated genes is provided in Table S12).

## 3. Results

### 3.1. The Disruption of Core-Clock Genes Alters the Rhythmic Expression of Genes Involved in Both Cancer- and Neurodegeneration-Related Pathways

**Figure 1:**
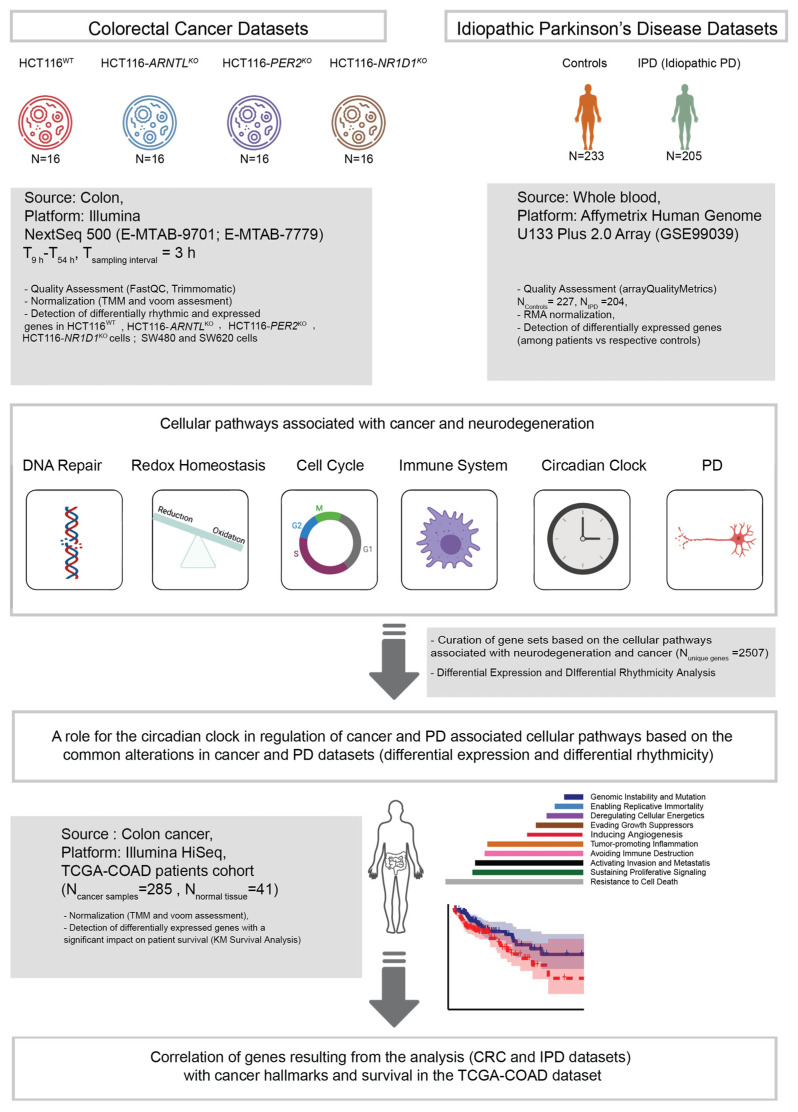
Workflow for the analysis of time-series RNA-seq data sets from CRC cell lines and microarray data sets from a cohort of IPD patients. KO cells were derived from HCT116^WT^ cells using CRISPR/Cas9 methodology. RNA-seq data sets (ArrayExpress: E-MTAB-9701) include the HCT116^WT^ and three core-clock knockout cells (HCT116-*ARNTL*^KO^, HCT116-*PER2*^KO^ and HCT116-*NR1D1*^KO^) sampled as indicated. Data sets of two additional CRC cell lines (SW480, SW620; ArrayExpress: E-MTAB-7779) were also included in the rhythmicity analysis as indicated. Following data pre-processing, circadian and ultradian rhythms were detected and differential rhythmicity analysis was performed. In addition, differential gene expression analysis was carried out for all expressed genes as compared to the corresponding controls, for HCT116 cells and IPD data sets (GEO: GSE99039).

Cancer and neurodegeneration share altered cellular processes including disruption of cell cycle, DNA repair, immune system and redox homeostasis [3,17,86]. To investigate possible circadian clock-related alterations in the expression of elements of these pathways, we gathered 438 microarray data sets retrieved from whole blood samples of IPD patients [54] (GEO: GSE99039), 64 RNA-seq data sets from CRC cell lines (newly generated data sets for HCT116^WT^ and three HCT116 derived-KO cells (ArrayExpress: E-MTAB-9701) and previously generated 22 RNA-seq time course data sets for two additional CRC cell lines originated from the primary tumour and metastasis site of the same patient (SW480, SW620; ArrayExpress: E-MTAB-7779) and known to have different clock phenotypes [63]. The PD data (GEO: GSE99039) was obtained from whole blood of 205 patients (90 females, 101 males and 14 samples without sex information) with IPD and 233 controls (142 females, 70 males and 21 samples without sex information) (Figure 1, right panel and Table 1). We complemented the IPD data sets with newly generated data sets from three core-clock KO mutants (HCT116-*ARNTL*^KO^, HCT116-*PER2*^KO^ and HCT116-*NR1D1*^KO^) and the corresponding WT cells, as well as previously published data sets from two CRC cell lines as indicated above, to investigate possible clock-related alterations relevant in cancer development and neurodegeneration-related genes with a particular focus on PD. For all four cell lines (HCT116^WT^ and HCT116-derived KOs), we generated RNA-seq time course data (Figure 1, left panel).

For the time-course data sets, we performed a rhythmicity analysis for genes oscillating with circadian (24 h) and ultradian periods (8 h and 12 h) and a subsequent differential rhythmicity analysis to characterize KO-related variations in the rhythmic expression phenotype (loss of oscillations or change in phases and/or amplitudes). In addition, and to allow for data comparison between the IPD (single time point data sets) and the CRC data (time-course data sets), we performed a differential expression analysis to investigate putative alterations in the mean gene expression levels in the HCT116 cells and the PD data.

We focused our analysis on molecular mechanisms, commonly disrupted in both cancer and PD-related pathways. For this, we curated a gene list based on a literature search on commonly disrupted pathways in both cancer and neurodegeneration with a focus in PD, which included several KEGG pathways, namely immune system (Table S2), DNA damage (Table S3), cell cycle (Table S4), redox homeostasis (from energy metabolism and catabolism pathways) (Table S5), a published list of core-clock genes comprising the core-clock network (CCN) and the ECCN [87,88], (Table S6), and the PD pathway (Table S7). The last one was complemented based on a literature curation of published studies describing additional genes involved in genetic forms of PD or linked to increased risk of symptoms with possible circadian influences (motor fluctuations, hallucinations and psychosis, sleep symptoms including REM behavior disorder or restless legs syndrome) (Table S7). This resulted in 2507 unique genes, named hereafter as “genes of interest”.

**Figure 2:**
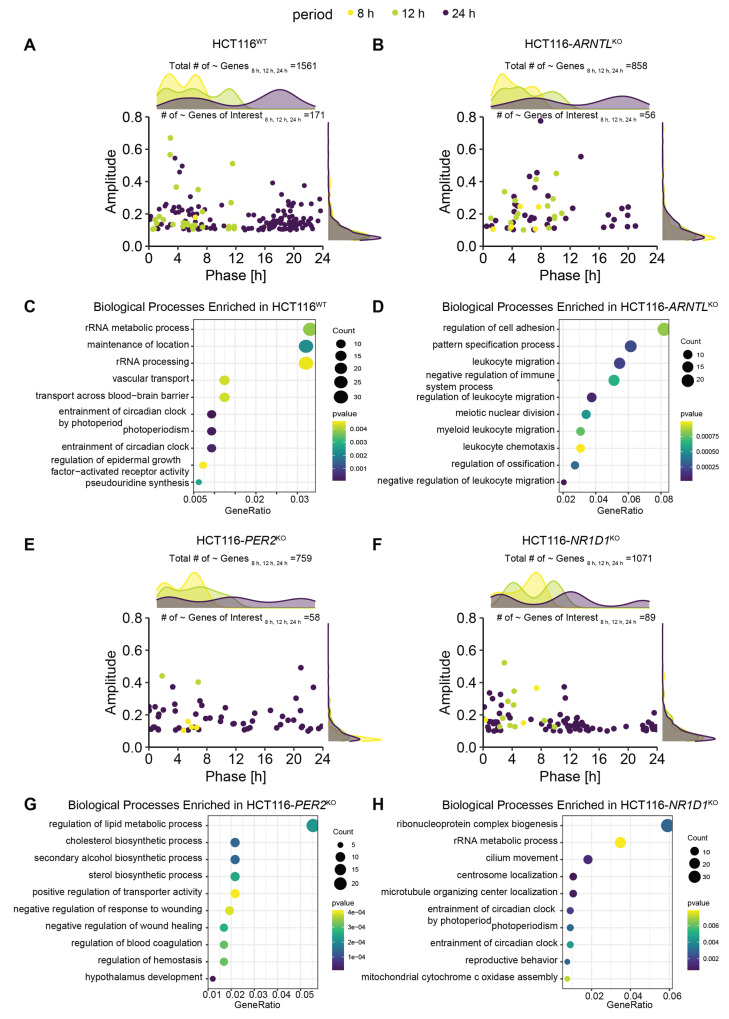
Core-clock KO affects the rhythmic characteristics of expressed genes in HCT116 cells and the associated biological processes. (**A**) Acrophase and amplitude distribution of significantly oscillating genes (based on RAIN *q* < 0.05 and relative amplitude ≥ 0.1) in HCT116^WT^ cells (N_total_~_8 h,12 h,24 h_ = 1561,N_total24 h~genes_ = 1241, N_total12 h~genes_ = 273, N_total8 h~genes_ = 46; N_totalGenesofInterest_~_8 h,12 h,24 h =_ 171, N_24 h~GenesofInterest_ = 143, N_12 h~GenesofInterest_ = 27 and N_8 h~GenesofInterest_ = 1), (**B**) Acrophase and amplitude distribution of significantly oscillating genes in HCT116-*ARNTL*^KO^ (N_total_~_8 h,12 h,24 h_ = 858, N_total24 h~genes_ = 459, N_total12 h~genes_ = 321, N_total8 h~genes_ = 78; N_totalGenesofInterest_~_8 h,12 h,24 h =_ 56, N_24 h~GenesofInterest_ = 35, N_12 h~GenesofInterest_ = 16; N_8 h~GenesofInterest_ = 5), (**C**) Top 10 GO (Biological Processes) enrichment results of 24 h rhythmic genes in HCT116^WT^ cells (**D**) Top 10 GO (Biological Processes) enrichment results for 24-h rhythmic genes in HCT116-*ARNTL*^KO^ (**E**) Acrophase and amplitude distribution of significantly oscillating genes in HCT116-*PER2*^KO^ (N_total_~_8 h,12 h,24 h_ = 759, N_total24 h~genes_ = 539, N_total12 h~genes_ = 179, N_total8 h~genes_ = 41; N_totalGenesofInterest_~_8 h,12 h,24 h =_ 58, N_24 h~GenesofInterest_ = 52, N_12 h~GenesofInterest_ = 2; N_8 h~GenesofInterest_ = 4) (**F**) Acrophase and amplitude distribution of significantly oscillating genes in HCT116-*NR1D1*^KO^ (N_total_~_8 h,12 h,24 h_ = 1071, N_total24 h~genes_ = 884, N_total12 h~genes_ = 152, N_total8 h~genes_ = 35; N_totalGenesofInterest_~_8 h,12 h,24 h =_ 89_,_ N_24 h~GenesofInterest_ = 76, N_12 h~GenesofInterest_ = 10 and N_8 h~GenesofInterest_ = 3) (**G**) Top 10 GO (Biological Processes) enrichment results for 24-h oscillating genes in HCT116-*PER2*^KO^ (**H**) Top 10 GO (Biological Processes) enrichment results for 24-h oscillating genes in HCT116-*NR1D1*^KO^. Density plots on top panels indicate total number of oscillating genes. Number of oscillating genes of interest (for 8 h, 12 h, and 24 h periods) and total number of oscillating genes (for 8 h, 12 h, and 24 h periods) indicated above each panel. Pre-selected genes of interest are visualized in the scatter plots. The genes oscillating with different periods are colour coded (24 h^~^, purple; 12 h^~^, green; 8 h^~^, yellow). “~” stands for significantly oscillating genes.

To study the influence of clock dysregulation on the genes of interest, we performed a rhythmicity analysis in HCT116^WT^ and the three KO cell lines. As expected, for the KO cells we observed a reduction in the overall number of rhythmic genes (both circadian and ultradian). In addition, we observed a variation in the global phase distribution in all HCT116^KO^ conditions when compared to HCT116^WT^ (Figure 2A,B,E,F, density plots). HCT116^WT^ cells showed a bimodal distribution for genes expressed with 8-h and 24-h periods, whereas a multimodal distribution was observed for genes expressed with a period of 12 h, and this pattern was lost in all KOs (Figure 2A,B,E,F). For the genes of interest, we plotted the phase and amplitude of rhythmic genes in each KO cell line (Figure 2A,B,E,F, scatter plots). All three KOs led to a decrease in the number of rhythmic genes (within the set of genes of interest), as expected (HCT116^WT^: N_24h~GenesofInterest_ = 143, N_12h~GenesofInterest_ = 27 and N_8h~GenesofInterest_ = 1; HCT116-*ARNTL*^KO^: N_24h~GenesofInterest_ = 35, N_12h~GenesofInterest_ = 16; N_8h~GenesofInterest_ = 5; HCT116-*PER2*^KO^: N_24h~GenesofInterest_ = 52, N_12h~GenesofInterest_ = 2, N_8h~GenesofInterest_ = 4; HCT116-*NR1D1*^KO^: N_24h~GenesofInterest_ = 76, N_12h~GenesofInterest_ = 10 and N_8h~GenesofInterest_ = 3) (Figure 2A,B,E,F). Most of the rhythmically expressed genes of interest were circadian expressed (66%), whereas only 33% showed 12 h rhythms, in HCT116^WT^ cells. Out of the rhythmic set of genes of interest, only 1% had 8 h periods in HCT116^WT^ cells. In all KO conditions, a similar pattern was observed in the proportion of rhythmic genes of interest oscillating with different periods. The majority of genes oscillated with a 24 h period.

Moreover, the oscillatory characteristics of the genes of interest depicted in the scatter plots (amplitudes, and phases estimated by the rhythmicity analysis) resembled those observed in the density plots for the total number of oscillating genes, with a bimodal distribution of phases for 24 h rhythmic genes in HCT116-*ARNTL*^KO^ cells (Figure 2B) and a multimodal distribution of phases (0–8 h; 8–16 h; 16–24 h) in HCT116-*PER2*^KO^ and HCT116-*NR1D1*^KO^ cells (Figure 2E,F). This indicates that our pre-selected genes of interest show alterations in rhythmicity upon core-clock manipulation, which reflect the global expression changes in each KO cell line.

To identify highly represented biological processes for rhythmic genes in HCT116^WT^ and KO conditions, we performed an enrichment analysis using the 24-h (Figure 2C,D,G,H) and 12-h rhythmic genes (Figure S2). The top 10 GO biological process in HCT116^WT^ and in HCT116-*NR1D1*^KO^ showed an enrichment of genes involved in entrainment of circadian rhythms and RNA processing (Figure 2C,H). In HCT116-*ARNTL*^KO^ (Figure 2D), the majority of top enriched biological processes were related to immune system and cell motility, whereas in HCT116-*PER2*^KO^ (Figure 2G) metabolic processes and angiogenesis pathways were among the top listed enriched biological pathways. For 12-h rhythmic genes, our analysis showed an enrichment of metabolic processes in HCT116^WT^ (Figure S2A) and HCT116-*NR1D1*^KO^ (Figure S2D), which are processes known to be rhythmic with shorter periods [33,61], whereas developmental processes were enriched in HCT116-*ARNTL*^KO^ (Figure S2B) and HCT116-*PER2*^KO^ (Figure S2C).

**Figure 3:**
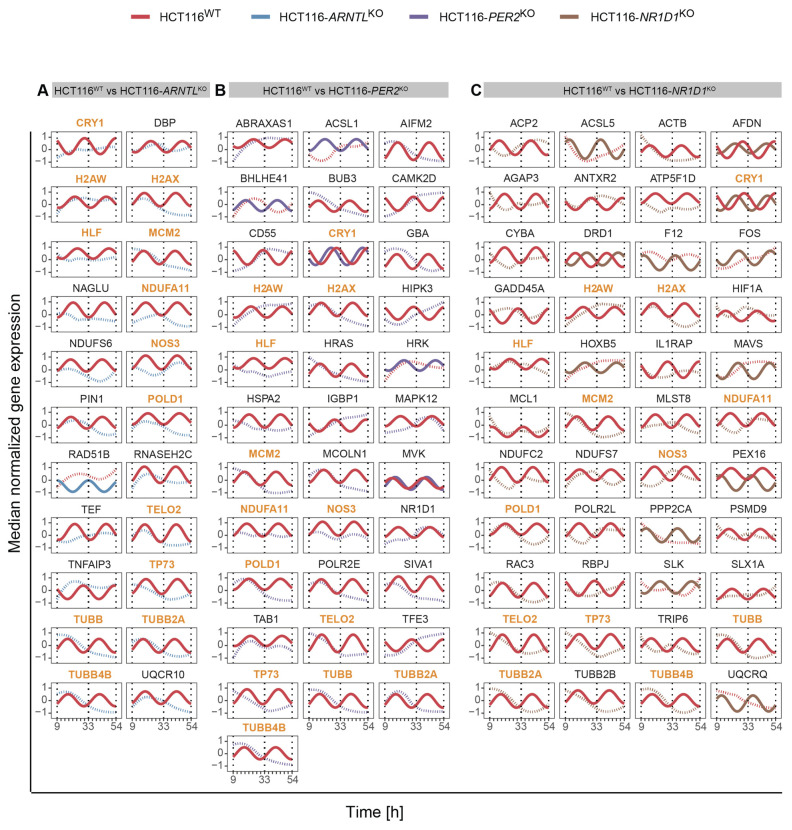
Circadian expression profiles of genes of interest detected as differentially rhythmic in all HCT116 KOs. (**A**) in HCT116^WT^ and HCT116-*ARNTL*^KO^ (**B**) in HCT116^WT^ and HCT116-*PER2*^KO^ (**C**) in HCT116^WT^ and HCT116-*NR1D1*^KO^. Significantly rhythmic genes (*q* < 0.05; relative amplitude ≥ 0.1) are depicted using a harmonic regression curve (full lines); not significantly rhythmic genes are depicted with LOESS (Locally Estimated Scatterplot Smoothing) (dashed lines) (see Figure S3 for similar analysis in SW480 and SW620 cells). Common differentially rhythmic genes across all KO cells, as compared to the WT, are marked in orange.

We further evaluated the influence of clock disruption on the overall rhythmicity profiles of the genes of interest for each KO versus the WT cells (Figure 3A–C). For this, we carried out a differential rhythmicity analysis for all genes oscillating in at least one of the conditions (WT or KO) with 24- and 12-h periodicity. No differentially rhythmic genes were found with eight-hour periods. Among the circadian genes, 13 differentially rhythmic genes were identified in all KO conditions as compared to the WT. These included the core-clock gene *CRY1*, genes crucial for transcriptional regulation, DNA repair and cell growth including *HLF* (transcription factor, related to the PAR BZIP family and associated with leukaemia)*, POLD1* (DNA polymerase associated with colorectal cancer), *MCM2* (mini-chromosome maintenance protein regulating DNA replication), *TP73* (a member of TP53 family and a candidate gene for neuroblastoma) in which one of the isoforms, ΔNp73, is known to function as a pro-survival factor in neurons whose haploinsufficiency might be a risk factor for neurodegeneration [89,90,91], histone protein variants *H2AW* and *H2AX, TELO2* (S-phase checkpoint regulator), and important regulators of redox homeostasis like *NOS3,* which acts as a mediator in several biological processes, including neurotransmission and antitumoral activities, *NDUFA11* involved in the mitochondrial electron transport chain, and microtubules structural components like *TUBB, TUBB2A, TUBB4B*, which regulate intracellular transport and neuronal communication (Figure 3A–C). These genes depicted significant oscillations in expression in HCT116^WT^ cells, whereas their rhythmicity was disrupted in the KO cells. The KO of *ARNTL* led to the strongest effect with loss of rhythmicity for all genes listed above (Figure 3A). In addition, each KO showed a unique set of differentially rhythmic genes of interest. Among the differentially rhythmic genes unique to HCT116-*ARNTL*^KO^ were the transcriptional regulator genes *DBP* and *TEF*, which showed a disruption of rhythmicity, mitochondrial- and lysosomal-energy-metabolism-related genes *NDUFS6*, *NAGLU*, *UQCR10,* genes related to cellular growth and DNA repair mechanisms *RAD51B, RNASEH2C,* inhibitor of TNF-induced apoptosis gene *TNFIAP3,* and *PIN1*, which regulates genotoxic and other stress responses, as well as cellular growth (Figure 3A). The majority of differentially rhythmic genes specific to HCT116-*PER2*^KO^ are involved in the regulation of DNA damage response, cell cycle and apoptosis (*ABRAXAS1, AIFM2, BUB3, HIPK3, HRAS, HRK, MAPK12, SIVA1, TAB1* and *TFE3*) (Figure 3B). Moreover, two well-known clock genes, *NR1D1* and *BHLHE41* (also known as *DEC2*, a clock gene that interacts with ARNTL or competes for E-box binding sites in the promoter region of *PER1*) were also differentially rhythmic uniquely in HCT116-*PER2*^KO^ (Figure 3C). In addition, genes involved in the regulation of energy metabolism such as *GBA* (a lysosomal membrane protein involved in glycolipid metabolism), which is found to be frequently mutated in PD patients and linked to increased disease susceptibility, *ACSL1, CAMK2D, MCOLN1* and *MVK*, genes involved in immune system activation and immune cell differentiation such as *CD55* and *IGBP1*, the transcription regulator *POLR2E*, which encodes for RNA polymerase and *HSPA2*, a regulator of protein synthesis (protein folding, transport and proteolysis of misfolded proteins), were also identified to be differentially rhythmic only in HCT116-*PER2*^KO^. Most of the differentially rhythmic genes unique to HCT116-*NR1D1*^KO^, were involved in energy metabolism and regulation of ubiquitination (*ACP2, ACSL5, AGAP3, ATP5F1D, HIF1A, NDUFC2, NDUFS7, PEX16, PSMD9, UQCRQ*). In addition, several genes responsible for the regulation of genomic stability (apoptosis and DNA repair) and regulators of cellular proliferation (*FOS, GADD45 MCL2, MLST8, PPP2CA, RAC3, RRBPJ, SLK, SLX1A*) were differentially expressed (Figure 3C). Strikingly, *DRD1*, which encodes for the most abundant dopamine receptor in the central nervous system (CNS), was also differentially rhythmic in this KO, showing a phase shift between HCT116^WT^ and HCT116-*NR1D1*^KO^ (Figure 3C). Additionally, genes involved in regulation of cellular senescence (*CYBA*), cellular signalling (*ACTB, AFDN*), cellular structural organization (*ANTXR2, RAC3* and *TRIP6*), transcriptional regulator of polymerase II (*POLR2L*), immune system activity (*IL1RAP, MAVS*), *F12* (coagulation Factor XII), which functions as activator of the blood clotting cascade, as well as *TUBB2B* (major component of microtubules) were found to be uniquely differentially rhythmic in HCT116-*NR1D1*^KO^ (Figure 3C). As a comparison, we additionally analysed the expression profiles of the same genes in the CRC cell lines SW480 and SW620 and also observed variations in their circadian expression profiles (Figure S3). Taken together, these results suggest that the manipulation of core-clock genes results in alterations of rhythmicity patterns of genes involved in cancer and neurodegeneration.

### 3.2. The Impact of Core-Clock Knockouts in CRC Cells Shows an Overlap of Differentially Expressed Genes with the Idiopathic Parkinson’s Disease Cohort

**Figure 4:**
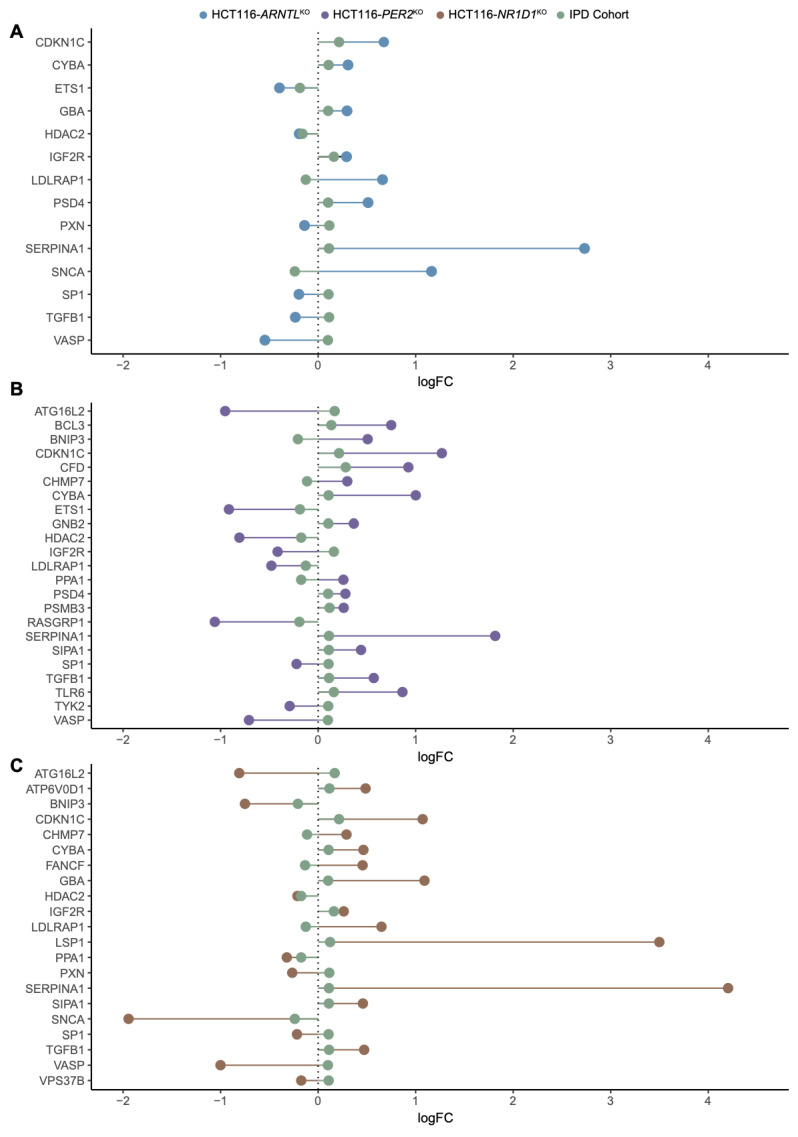
Common differentially expressed genes of interest in HCT116 KO cells and IPD cohort. Lollipop plots show the direction of changes (up/downregulation) in mean gene expression levels (log_2_ FC in comparison to respective controls) for the genes of interest shared between (**A**) HCT116-*ARNTL*^KO^ versus HCT116^WT^ and IPD patients versus controls (**B**) HCT116-*PER2*^KO^ versus HCT116^WT^ and IPD patients versus controls (**C**) HCT116-*NR1D1*^KO^ versus HCT116^WT^ and IPD patients versus controls.

To further explore the overlap between gene expression alterations in blood-derived IPD datasets and our CRC model, we performed a differential expression analysis comparing mean gene expression levels of HCT116^KO^ to WT cells and IPD patients to a control group of healthy subjects. For IPD data sets, we divided the cohort into females’ and males’ subgroups to also detect possible sex-specific effects. We then performed an enrichment analysis to identify biological processes associated to the differentially expressed genes (Figures S4 and S5). Our results showed that both in the HCT116^KO^ cells, as well as in the cohort of IPD patients, the majority of top represented biological processes were related to immune system functioning (Figures S4 and S5). In addition, the observed expression changes for the genes of interest, which were commonly differentially expressed in all core-clock KOs, suggested the existence of a KO specific effect in the regulation of these genes (Figure S6). Following our analysis, we identified commonly differentially expressed genes of interest in each core-clock KO cell line and in the IPD cohort versus the respective controls (Figure 4A–C). HCT116-*PER2*^KO^ shared the highest number of genes with the same variations in a synergistic way (upregulation/downregulation) as in the IPD cohort followed by HCT116-*NR1D1^KO^* and HCT116-*ARNTL^KO^*. On the contrary, HCT116-*PER2*^KO^ and HCT116-*NR1D1*^KO^ showed the same and also the highest number of antagonistic changes (inverse gene expression alteration compared to the IPD data sets) followed by HCT116-*ARNTL^KO^*.

Next, we extracted commonly differentially expressed genes between the IPD and the KO cells. Out of these genes, *PXN*, a mediator of extracellular matrix organization and focal adhesion, and one of our genes of interest, was differentially expressed in both HCT116-*NR1D1^KO^* and HCT116-*ARNTL^KO^* cells and in the IPD cohort (Figure 4A–C). *PXN* is known to promote tumour progression in cervical cancer [92]. Additionally, *PSMB3, SIP3* and *TYK2* (unique to HCT116-*PER^KO^)* and *SIPA1, ATP6V0D1* (differentially expressed only in HCT116-*NR1D1*^KO^) were also found within the top differentially expressed genes of interest in the IPD cohort. Among the differentially expressed genes of interest, nine genes showed a change in expression levels in all KOs and IPD patients. These included *CYBA* and *LDLRAP1s*, involved in oxidase activity and endocytosis, *VASP* involved in axonal guidance and structure, *IGF2R, SERPINA1, HDAC2, TGFB1* involved in immunity and inflammation and *CDKN1C* and *SP1* involved in cell cycle and growth (Figure 4A–C). *LSP1* (intracellular F-actin binding protein regulating adhesion, motility and migration)*, ATP6V0D1* (a component of vacuolar ATPase mediating acidification of intracellular organelles)*, FANCF* (Fanconi anaemia complementation group F) and *VPS37B* (vacuolar protein sorting involved in calcium-dependent protein binding) were differentially expressed in the IPD cohort and HCT116-*NR1D1*^KO^ cells (Figure 4C). *CHMP7* (charged multivesicular body protein seven involved in cellular senescence)*, PPA1* (member of the inorganic pyrophosphatase (PPase) family involved in phosphate metabolism of cells)*, ATG16L2* (autophagy related protein), *BNIP3* (mitochondrial protein that contains a BH3 domain and acts as a pro-apoptotic factor) and *SIPA* (mitogen induced GTPase activating protein (GAP) involved in RAS pathway and innate immune system) were differential expressed in HCT116-*PER2*^KO^ cells and the IPD cohort (Figure 4B), whereas no differentially expressed genes were found common to the IPD cohort and solely unique to the HCT116-*ARNTL^KO^* cells.

**Figure 5:**
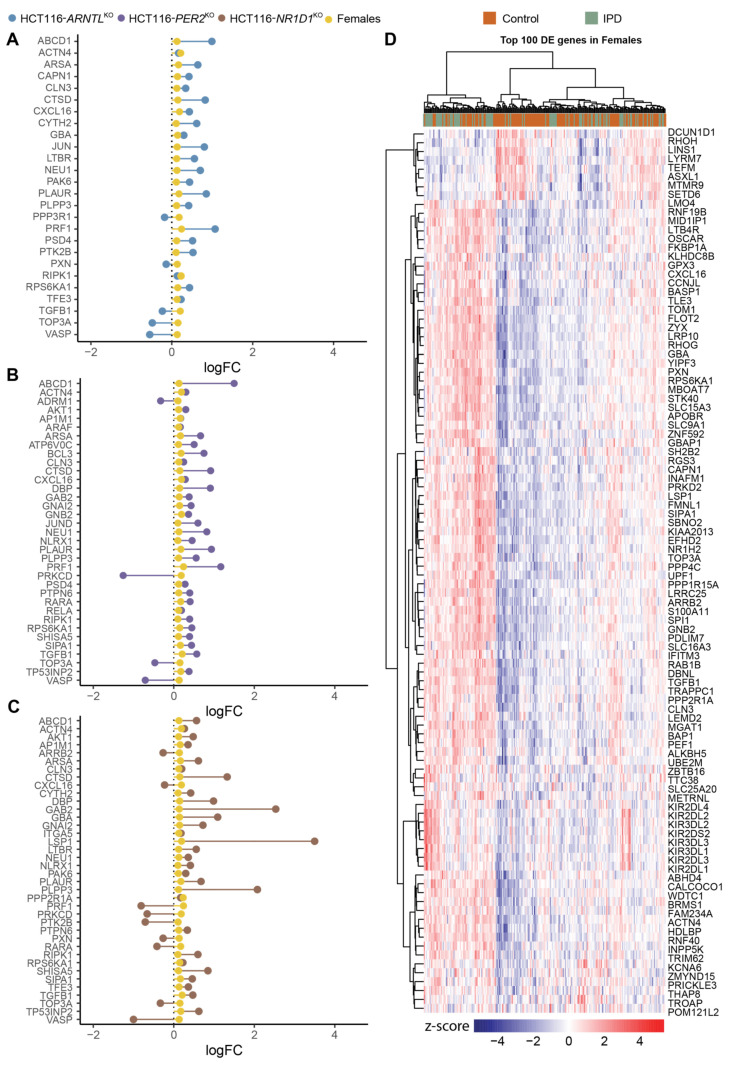
Commonly differentially expressed genes of interest in HCT116 KOs and female IPD patients. Lollipop plots show the direction of changes (up/downregulation) in mean expression levels (log_2_ FC in comparison to respective controls) for the genes of interest shared between (**A**) HCT116-*ARNTL*^KO^ versus HCT116^WT^ and female IPD patients versus respective controls; (**B**) HCT116-*PER2*^KO^ versus HCT116^WT^ and female IPD patients versus respective controls; (**C**) HCT116-*NR1D1*^KO^ versus HCT116^WT^ and female IPD patients versus respective controls. (**D**) Heatmap of top 100 differentially expressed genes (sorted by *p*-value) for IPD female patients. The heatmap was clustered using WardD2 linkage and Euclidean distancing. Disease status is depicted as orange for controls and green for patients. Colour code scheme for standardized gene expression values (z-scores) are indicated in the heatmap colour key (see Figures S7–S9 for similar analysis in males).

We next analysed the commonly differentially expressed genes in the KO cells and the IPD cohort following sex separation (N_IPD Females_ = 90, N_IPD Males_ = 100 and N_Control Females_ = 138, N_Control Males_ = 69). The subsequent differential expression analysis resulted in less differentially expressed genes shared between KO conditions and IPD cohort for females (Figure 5A–C) as compared to males (Figures S7 and S8). *CAPN1* and *JUN,* involved in apoptosis, and *PPP3R1,* involved in the immune system, were found to be differentially expressed specifically in HCT116-*ARNTL*^KO^ and in the female patients (Figure 5A). *GNB2* (guanine nucleotide-binding protein involved in ERK signalling)*, JUND* (member of the JUN family regulating p53-dependent senescence and apoptosis)*, RELA* (*p65*), a proto-oncogene and a subunit of the classical NF-κB cascade that forms a heterodimeric complex with the p50 (NF-κB1) subunit, *ARAF* (proto-oncogene and a member of the RAF subfamily involved in cell growth and development), *ADRM1* (adhesion regulating molecule 1 protein family regulating cell adhesion), *BCL3* (a proto-oncogene candidate and a transcriptional co-activator that activates through its association with NF-κB) were differentially expressed in the female patients and in HCT116-*PER2*^KO^ cells (Figure 5B), whereas *LSP1* (intracellular F-actin binding protein, regulating motility, adhesion and migration)*, ARRB2* (member of arrestin/β-arrestin protein family, involved in cellular responses to stimuli)*, PPP2RIA* (subunit of protein phosphatase two, implicated in the negative control of cell growth and division) and *ITGA5* (integrin α-chain family member, which functions in cell surface adhesion and ERK signalling) were differentially expressed in both HCT116-*NR1D1*^KO^ cells and the female IPD patients (Figure 5C). In addition, the core-clock regulator *DBP* was found to be commonly differentially expressed in HCT116-*PER2*^KO^, HCT116-*NR1D1*^KO^ and female patients (Figure 5B,C). Male IPD patients showed a higher number of differentially expressed genes of interest overlapping with the KO cells; however, more genes showed an opposite trend in their expression change compared to the KO cells, as in the female patients (Figures S7 and S8). We next analysed the top 100 differentially expressed genes in females and males. These genes did not provide a distinct genetic signature to fully distinguish controls from IPD patients (Figure 5D and Figure S9). Nevertheless, a different genetic signature seems to be associated with either IPD female or male patients.

### 3.3. The Relative Expression among ECCN Elements Varies in IPD Patients

**Figure 6:**
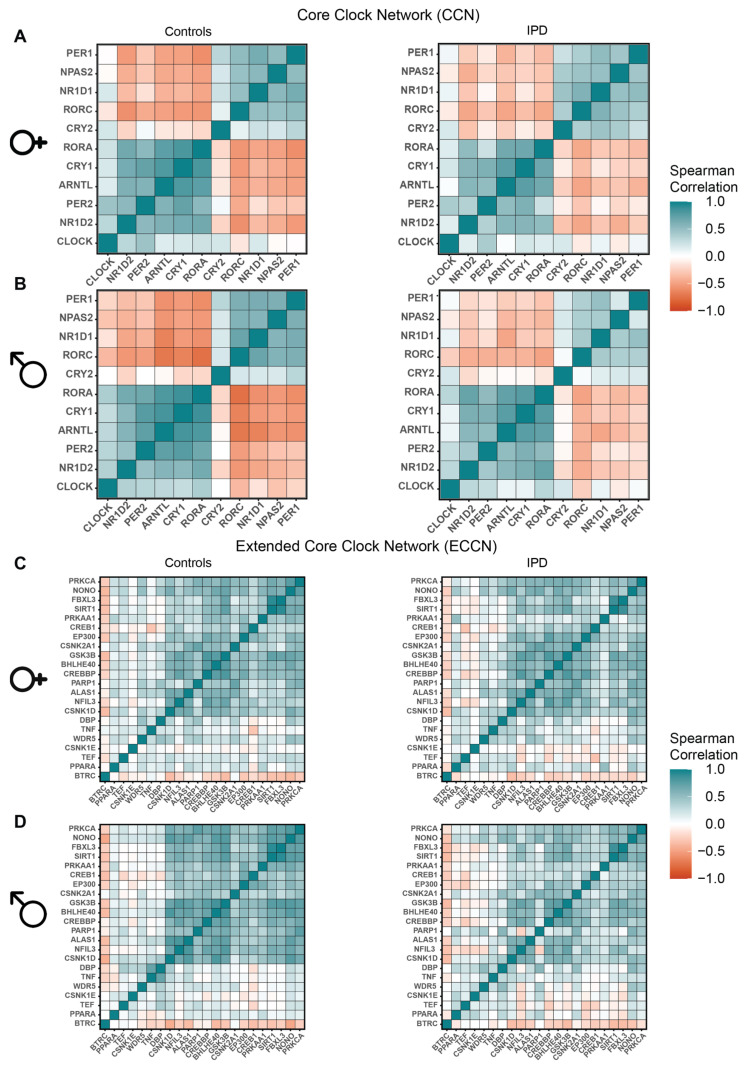
Correlation heatmaps of CCN and ECCN genes in IPD patients versus controls (sex separated). (**A,B**) Heatmap visualization of Spearman correlation between CCN genes in IPD, as well as the controls for (**A**) females and (**B**) males. (**C,D**) Heatmap visualization of Spearman correlation between each pair of the ECCN genes in IPD as well as in the control group for (**C**) females and (**D**) males.

Next, we investigated potential alterations in the expression of CCN and ECCN elements in the IPD cohort. *DBP*, a clock gene and a member of the family of PAR BZIP transcription factors, was differentially expressed in the female patients versus the respective control group (*p* = 0.002, log_2_FC = 0.146, Figure S10). We could not find ECCN elements to be differentially expressed in the full cohort (Figure S11) nor in male patients (Figure S12). We additionally performed a correlation analysis for the CCN and the ECCN elements in the IPD cohort data sets. The determined Spearman correlation coefficient was compared with the reference correlation values for the CCN and ECCN genes in the control groups and visualized using correlation matrices ordered based on the pattern of the control male subjects, for comparison (Figure 6A–D). The IPD male and the female patients showed a weaker correlation for both the CCN and the ECCN elements, compared to the respective control groups, as indicated by the Spearman coefficients (Figure 6A–D). To avoid redundancy between elements of the CCN and of the ECCN gene list, only the genes which are not part of the CCN were visualized in the ECCN correlation heatmaps (Figure 6C,D). Within the CCN, one set of genes (*CLOCK, NR1D2, PER2, ARNTL, CRY1, RORA*) showed positive correlation within the group, and negative correlation with the other set of genes (*CRY2, RORC, NR1D1, NPAS2, PER1*) in the IPD and control group. For the ECCN, *CSNK1D, NFIL3, ALAS1, PARP1, CREBBP, BHLHE40, GSK3B, CSNK2A1, EP300, CREB1, PRKAA1, SIRT1, FBXL3, NONO,* and *PRKCA* showed the strongest positive correlation within the group, and weaker or negative correlation with the second set of genes (*PPARA, TEF, CSNK1E, WDR5, TNF, DBP*) in the IPD and in the control groups (Figure 6C,D). *BTRC* showed negative correlation with all other genes within the ECCN, both for IPD patients as well as for the control groups. Although the pattern of correlation was qualitatively similar among the different groups, there was a weaker correlation among ECCN elements as suggested by the overall decrease in the Spearman correlation coefficients for the cohort of IPD patients (both for males and females) compared to the respective controls, which points to a dysregulation of the circadian clock network associated with the disease. This data also highlights the need for investigating the role for the circadian clock in IPD by generating time-course data sets to monitor possible alterations in the circadian expression of relevant genes in IPD progression.

### 3.4. Genes of Interest Exhibiting Differential Expression in Both CRC Cells and the IPD Cohort Show Alterations in Their Rhythmic Phenotype upon KO of Core-Clock Genes and Impact Survival in Colon Cancer Patients

**Figure 7:**
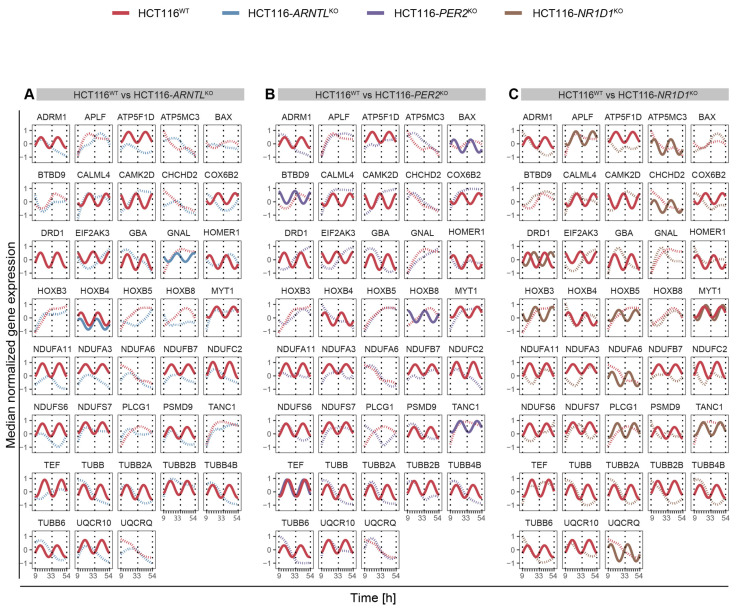
Circadian expression profiles of PD-associated genes. (**A**) in HCT116^WT^ and HCT116-*ARNTL*^KO^ (**B**) in HCT116^WT^ and HCT116-*PER2*^KO^ (**C**) in HCT116^WT^ and HCT116-*NR1D1*^KO^. Rhythmicity analysis was computed considering a 24-h period. Depicted are significantly rhythmic genes (*q* < 0.05 and relative amplitude ≥ 0.1) plotted using a harmonic regression fitting (full lines), whereas not significantly rhythmic genes were represented with LOESS (dashed lines) (see Figure S3 for similar analysis in SW480 and SW620 cells).

To characterize the potential effect of clock dysregulation in the rhythmicity patterns of the top differentially expressed genes in the IPD cohort, we selected, out of the set of genes of interest, the ones present in the list of the top 100 differentially expressed genes (sorted according to *p*-value) in the full cohort (without sex separation), as well as in the top 100 differentially expressed genes (sorted according to *p*-value) in female and male patients. Because the IPD data set was collected at a single time point, we used the HCT116 time course data sets to visualize possible rhythmic variations in gene expression. Within the IPD female patients, *GBA*, a crucial regulator of lysosomal functioning, was found to be among the most common genetic factors in IPD patients [55,93] and was circadian expressed in HCT116^WT^ cells. Mutations of *GBA* gene are linked with increased IPD incidence. Upon clock disruption, *GBA* lost circadian rhythmicity in all KO conditions (Figure S13, middle panel). Among the top differentially expressed genes in the IPD male cohort, *CYBA* gene was circadian expressed only in the HCT116^WT^ condition, whereas in all KO conditions, its rhythmicity was lost (Figure S13, bottom panel). *CYBA* functions as a regulator of oxidative stress, phagocytosis and immunity, processes known to be disrupted in PD. In addition, we have analysed the circadian profiles of the PD-related gene subset within the genes of interest (Table S7) that also showed alterations in their rhythmicity patterns (loss or gain of oscillations or a change in amplitude or phases) upon disruption of core-clock genes in CRC cells (Figure 7A–C and Figure S3). The genes showing an alternation in one of the parameters in either of the KO cells were visualized in all conditions in order to allow for a comparison between different core-clock KOs. *DRD1* showed an alteration in its rhythmicity in HCT116-*PER2*^KO^ (Figure 7B) and HCT116-*NR1D1*^KO^ (Figure 7C) compared to WT cells. *DRD1* was not expressed (based on our expression cutoff) in *ARNTL*^KO^ cells (Figure 7A). Among the PD-associated genes, which showed a fluctuation in their rhythmicity, *GBA* and *ADRM1* were differentially expressed in females and *TUBB2A* was differentially expressed in male IPD patients. These results suggest a potential role for the circadian clock as a regulator of genes involved in PD-associated molecular mechanisms. Altogether our results show that several of the differentially expressed genes found in the cohort of IPD patients are likely to show circadian variations and to be altered via perturbations of the core-clock, which may explain some of the molecular and physiological alterations reported in PD.

**Figure 8:**
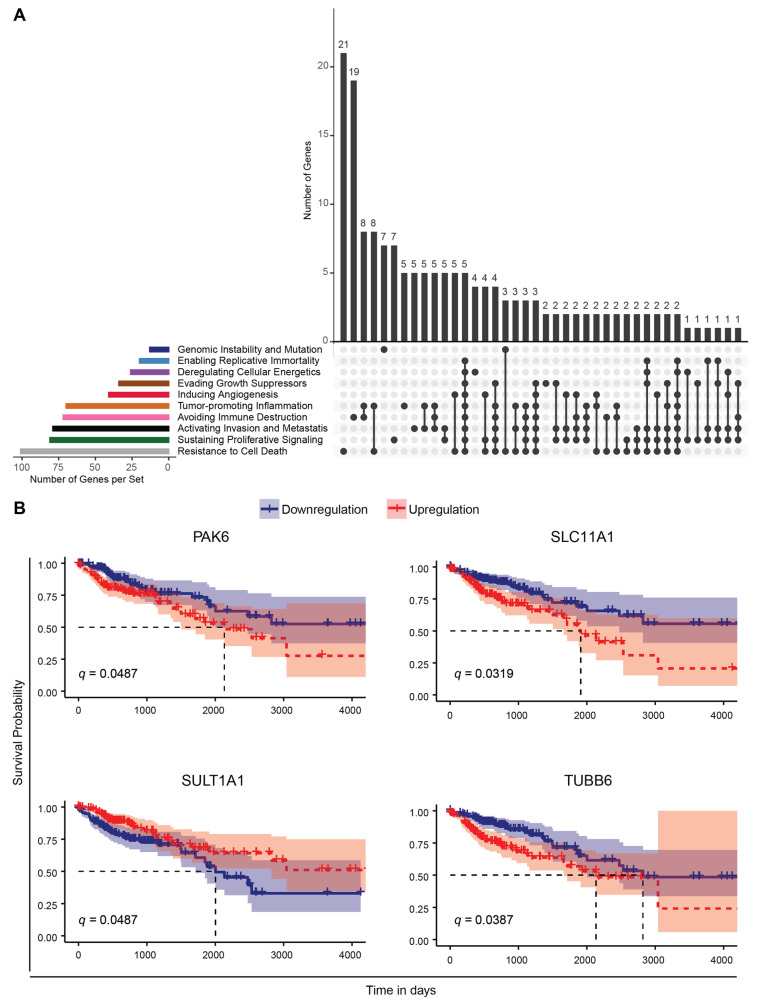
Correlation of genes resulting from the analysis with CRC cell lines and the cohort of IPD patients, with cancer hallmarks and survival in a colon adenocarcinoma cohort. (**A**) The plot depicts the intersection of cancer-hallmarks-related genes (retrieved from the CHG database [56]) and relevant genes highlighted by our analysis (Table S11). The colour coded bar plots (**left**) indicate the number of genes related to a particular cancer hallmark. The genes found to be associated with more than one hallmark are indicated with connecting lines (**right**). The number of unique genes and genes associated with more than one cancer hallmark are indicated above the grey bar plots (**right**). (**B**) Kaplan–Meier survival curves for genes found to be common in our analysis and genes associated with patient survival in the TCGA-COAD cohort. The patient cohort was separated into two groups based on the median gene expression level as high and low expression groups and a comparison of survival time (in days) was performed. In a subsequent analysis, using cancer stage as stratification of the patients, the following *q*-values were obtained: PAK6, *q* = 0.036; SLC11A1, *q* = 0.005; SULT1A1, *q* = 0.27; TUBB6, *q* = 0.01 (see also Table S12).

To investigate the significance of our results in colon cancer, we merged all relevant genes resulting from our analysis (473 unique set of genes, Table S11) and intersected with the cancer hallmarks genes extracted from the CHG (Cancer Hallmark Genes) database [30]. To do so, we selected the genes from our analysis according to the following criteria: (i) Genes exhibiting a differential rhythmicity in at least one of the KO cells (compared to WT); (ii) Common genes exhibiting differential expression in at least one of the HCT116^KO^ cells (compared to WT) and in the IPD cohort (sex separated, and also the full cohort without sex separation); and (iii) top 100 differentially expressed genes in the IPD cohort (sex separated, and also the full cohort without sex separation compared to respective controls). Our analysis revealed that 188 genes (about 40% of the total number of 473 genes short-listed based on our analysis) were associated with cancer hallmarks. The largest number of these genes were linked to “resistance to cell death”, followed by genes linked to “sustaining proliferative signaling” and “activation of invasion and metastasis” (Figure 8A).

Next, we performed a Kaplan–Meier survival analysis using TCGA COAD RNA-seq datasets, which revealed 289 genes out of the total number of genes found to be differentially expressed between cancer and normal tissue samples, to be significantly associated with patient survival. We found that four genes were also present in our analysis using the IPD cohort and CRC data sets, and were also part of the list of cancer-relevant genes retrieved from the CHG database [56]. These included the following: *TUBB6*, associated with ‘activation of invasion and metastasis’ and ‘tumour promoting inflammation’; *PAK6*, linked to ‘sustaining proliferative signaling’, ‘cell death resistance’, ‘activation of invasion and metastasis’, ‘evading growth suppressors’ and ‘immune destruction’; *SULT1A1*, linked to ‘genomic instability and mutation’, and *SLC11A1*, associated to ‘resistance to cell death’ (Figure 8B). *TUBB6*, which encodes for microtubules and has a crucial role in mitosis, intracellular transport and neuronal communication (motility and morphology), lost circadian rhythmicity in all KO cells (compared to WT). Downregulation of *TUBB6* expression resulted in increased survival probability in the TCGA-COAD cohort (Figure 8B). The differential expression of *PAK6*, which encodes for a member of p21-stimulated protein kinase family functioning in apoptosis and cytoskeletal rearrangement, was found to be significantly associated to patient survival in TCGA-COAD data and differentially expressed in *ARNTL*^KO^ and *NR1D1*^KO^ HCT116 cells, and in the IPD female patient cohort (Figure 8B). Downregulation of *PAK6* resulted in increased overall survival compared to high expression in the TCGA-COAD cohort. Among the top 100 differentially expressed genes in male IPD patients, *SULT1A1* encoding for a member of sulfotransferase enzyme family that catalyzes several hormones, neurotransmitters, as well as drug compounds and *SLC11A1* encoding for transporters involved in iron metabolism were found to be relevant for overall patient survival. Downregulation of *SULT1A1* resulted in a lower survival probability, whereas lower expression of *SLC11A1* yielded a higher survival probability in the TCGA-COAD cohort. Of note, additional clinical traits could be classified as a risk factor (e.g., age, sex, smoking, treatment) and may influence the outcome of survival analysis. While making an accurate assessment of all clinical traits remains a challenging task for the analysis of the data, we tried to address this issue by performing a comparative analysis using age, sex and disease stage as stratification factors and investigated the putative impact of these traits on patient overall survival. Among the clinical variables that we analyzed in the TCGA-COAD dataset, neither age at diagnosis nor sex were found to be significantly associated with survival (Figure S14A–D). Yet, cancer stage, was associated significantly with survival when the cohort was separated into four subgroups (Stage 1, 2, 3, 4) (Figure S14C). However, when subgrouping the cohort into early (1 and 2) and late (3 and 4) stage groups, we did not observe a significant impact of stage on the survival analysis (Figure S14). To further investigate the influence of the pre-selected differentially expressed genes on survival when considering cancer stage, we used a Cox Regression model to assess stage as a stratification factor for adjustment of the statistical testing (Table S12). Among the previously found cancer-hallmark survival-related genes (*PAK6*, *SLC11A1*, *SULT1A1* and *TUBB6*), we observed that their differential expression still had a significant impact on survival for three of the genes (*PAK6*, *SLC11A1* and *TUBB6*), when considering stage stratification. In addition, we identified four additional genes that were not included in the CHG database, but resulted from our analysis using IPD and CRC data sets and that showed a significant association with overall patient survival (Figure S15). These included *TRAP1*, which encodes for a molecular chaperone member of Hsp90 (Heat Shock Protein) family and was commonly differentially expressed in all HCT116^KO^ cells and in IPD male cohort; *FAM50B*, encoding for a protein associated with chromatin organization; *SPON2*, involved in cellular adhesion and embryonic neuronal outgrowth; *FES*, encoding for a tyrosine kinase that is essential for the maintenance of cellular transformation, were among the top 100 differentially expressed genes in the IPD cohort (without sex separation) and found to be significantly associated with survival in cancer patients (Figure S15). Interestingly, *FAM50B* was downregulated in HCT116-*ARNTL*^KO^, HCT116-*NR1D1*^KO^ and in male IPD patients, whereas it was upregulated in HCT116-*PER2*^KO^ cells, which points to an alteration in the expression of this gene both in IPD and as a result of core-clock modifications. Downregulation of *FAM50B*, *FES* and *SPON2* resulted in an increased survival probability, whereas upregulation of *TRAP1* was associated with higher survival probability. Altogether these findings suggest that several genes resulting from our analysis, altered in both CRC cells and IPD patients, are part of the cancer hallmarks gene sets and impact survival in colon cancer patients.

## 4. Discussion

PD is an intrinsically fluctuating disorder. As the disease progresses, patients describe symptomatic daily variations [94], commonly attributed to the administration timing of anti-parkinsonian drugs. Patients suffer from disabling off periods, as the dopaminergic drugs fade away, or, conversely, troublesome dyskinesias arise in synchrony to peak levels of these same drugs [95]. There are, however, fluctuations that are unrelated to drugs, and empirical clinical evidence suggests that some fluctuations follow a 24-h rhythm. As the disease progresses, patients report a progressive decrease in motor function late in the day [96,97], also known as “sundown syndrome”, and in more advanced PD stages, there is a common complaint of night-time akinesia, which affects over 70% of patients and prevents patients from changing sleep position or easily moving out of bed [98]. Contrary to this, some patients report an improvement in motor-, cognitive- and mood-related symptoms after waking up, even though they did not take regular medications during the night [99]. The motor response to repeated L-dopa administration also shows fluctuations during the day with weaker responses late in the evening and afternoon, as compared to the morning doses [100]. Visual discrimination deficits, which are frequently affected in PD, are also more prominent in evening hours [101]. Clinically, PD patients show blunted amplitudes in physiological variables that are expected to show diurnal variations. The rest-activity cycle measured through actigraphy shows decreased activity during the day in PD patients and higher movement levels during the night, resulting in an overall flattening of the circadian curve [102,103,104,105,106]. Studies on the variation of autonomic function throughout the day also suggest circadian changes in PD patients related to core body temperature profile [107,108,109], blood pressure [110] and heart rate variability [111,112]. Aziz et al. [113] evaluated the secretion profiles of growth hormones, thyroid-stimulating hormone, prolactin, leptin, adiponectin and resistin in de novo untreated PD patients and found no alterations. Cortisol, on the contrary, has also been shown to have an elevated secretion profile during the night in PD [50,114]. The dosage and timing of dopaminergic medications has been shown to increase melatonin secretion, another hormone with known circadian variations [115,116]. Melatonin, one of the important components of the circadian system, was shown to have a 24-h secretion profile significantly lower in PD patients, in a controlled design study using a modified constant routine [117] and in another sample of early PD patients [50]. Moreover, individual differences in melatonin profiles have been reported including a marked variability that occurs in PD in contrast to controls [116,118]. It is also possible that melatonin changes are not ubiquitous in PD because it is increasingly recognized that there are different subtypes of this disease [119]. In addition, sleep disturbances, one of the core clinical features of circadian dysfunction, are very common in PD, with different clinical manifestations ranging from insomnia to excessive daytime sleepiness [20,120,121]. The link between these observations from clinical data, physiological and molecular changes and circadian dysfunction is tempting, despite still-incomplete evidence.

### 4.1. Circadian Dysregulation of Cancer and PD-Related Pathways

Cancer development has also been linked with circadian dysregulation with several studies pointing to the role of core-clock genes as tumour suppressors [28,34,35,122,123]. Thus, and even though these two pathologies seem rather opposite in terms of cellular fate output [3,86], PD and cancer are both age-related diseases that share common disrupted pathways. According to our results, these shared altered pathways are likely circadian regulated. These commonly altered mechanisms include malfunctions in several processes including redox homeostasis, functioning of immune system, cell cycle, DNA repair and circadian regulation [3,6,13,14]. Our analysis shows that upon perturbation of core-clock genes, the rhythmicity pattern of genes involved in such processes was altered. According to our findings, the KO of *PER2*, which has previously been reported to act as a tumour-suppressor (reviewed in [124]), led to a drastic reduction in the number of oscillating genes compared to HCT116^WT^ cells and the strongest change in the phase distribution in all investigated periods. Differential rhythmicity analysis reflected loss of rhythmicity of lysosomal biogenesis and autophagy genes, which are known to be frequently disrupted in PD [125,126]. This subset of genes included *TFE3* and *MCOLN2* (altered in *PER2*^KO^ cells), *NAGLU* (altered in *ARNTL*^KO^ cells) and *PP2CA* and *ACP2* (altered in *NR1D1*^KO^ cells). *PPP2CA* encodes for the catalytic unit of phosphatase 2A (*PP2A*), which acts as a tumour suppressor [127]. It prevents WEE1 (G2/M checkpoint regulator) from proteolysis, thereby leading to elevated levels of WEE1 protein and subsequent cell cycle arrest, and thus results in an impairment of cellular proliferation (reviewed in [128]). PP2A was found to be downregulated in certain types of cancer (such as glioblastoma [129]) and neurodegenerative disorders including Alzheimer’s disease [130] and PD [131]. In PD, low activity of PP2A has been reported, and α-synuclein, a key component in the pathology of PD due to its abnormal accumulation, which results in Lewy bodies, acts as a regulator of PP2A. Based on our study, the abolished rhythmicity of *PP2CA* in *NR1D1*^KO^ can potentially suggest that its circadian variation might be regulated by *NR1D1*. A previous study in drosophila reported that PP2A regulates PER phosphorylation, thereby governing its activity in vitro and in vivo [132]. However, to the best of our knowledge, no studies have previously suggested a potential association between PP2A and the circadian clock in mammals. *NOS3*, an inhibitor of apoptosis and promoter of angiogenesis, lost circadian expression in all KOs compared to WT cells. *NOS3* was shown to play a pivotal role in cancer by promoting angiogenesis, metastasis formation and invasiveness, inhibition of apoptosis and immune response. Among metastatic colorectal cancer patients, *NOS3* inversely correlated with disease-progression-free survival and overall survival [133]. Interestingly, *NOS* isoforms (*NOS1*, *NOS2A*, and *NOS3*) are suggested to be strong candidates as susceptibility genes linked to environmental stress in PD [134]. Another important example of circadian regulated genes in both cancer and PD is *GADD45A*, a downstream mediator of p53, and regulator of the cell cycle and DNA repair. *GADD45A* acts as a tumour suppressor [135] and was found to be correlated with the expression of the core-clock gene *PER2*, in human colorectal carcinoma tissues [136]. In addition, in an in vitro model of PD, the knockdown of *GADD45A* was proposed to be neuroprotective against neuronal death induced by MPP (1-Methyl-4-phenylpyridinium), which is a metabolite used to mimic mitochondrial impairment in in vitro models of PD by inducing dopaminergic neuronal death [137]. Our results showed that *GADD45A* was differentially rhythmic in *NR1D1*^KO^ cells, as well as in SW480 versus SW620 cell lines, where we observed circadian rhythms of *GADD45A* only in the SW480 cells. Taken together, the variations in the circadian phenotypes of these genes upon core-clock perturbation point to a role for circadian regulation in genes which are involved in PD and cancer.

Mitochondria, the key component of energy metabolism, is tightly linked to several high ATP demanding neuronal processes such as axonal transport, due to its role in ATP production. Elevated lipid peroxide levels and mitochondrial malfunctioning has been previously shown in PD [138,139]. Strikingly, we observed a loss of rhythmicity of mitochondrial and oxidative phosphorylation genes of the NDUF family in our KO cell lines (*NDUFA11* in all KO conditions, *NDUFS7*, *NDUFC2* in *NR1D1*^KO^ cells and *NDUFS6* in *ARNTL*^KO^ cells). In addition, several clock genes were differentially rhythmic in the KO cells (*CRY1*, *NR1D1*, *DBP*, *TEF*, *BHLHE41*). Interestingly, *TEF* has been previously linked to a *TEF* variant associated with PD (rs738499) and found to be associated with sleep disturbances in PD [47]. Furthermore, the dopaminergic receptor *DRD1* was found to be differentially rhythmic in *NR1D1*^KO^ cells, and showed a phase delay compared to WT cells. Aberrant interactions between different dopamine receptors in the brain may be involved in L-dopa-induced dyskinesia [140] and risk of hallucination [141], and based on our findings circadian factors might also be involved in this interplay. Moreover, a regulator of vesicle trafficking, *LRRK2*, which is a frequently mutated gene in PD [142,143], has been shown to impair *DRD1* signalling transduction in mice [144].

### 4.2. Clock-Controlled Genes Are Differentially Expressed in PD

Our analysis indicated that some of the differentially rhythmic genes in the CRC data were also differentially expressed in the IPD cohort. These included genes linked to oxidative stress and energy metabolism such as *CYBA*, *TFE3*, *DBP* and *GBA*. *GBA* encodes for the lysosomal enzyme β-glucocerebrosidase and mutations in *GBA* are a key risk factor identified for patients with IPD [93,145,146]. *GBA* was upregulated in *ARNTL*^KO^ cells and *NR1D1*^KO^ cells, as well as in the IPD patients. Moreover, *GBA* was rhythmic in HCT116^WT^ cells, whereas its rhythmic expression was lost in all KOs. PD patients carrying *GBA* mutations show more dyskinesia, as well as other fluctuating symptoms like dysautonomia and hallucinations [147]. We observed that changes on the mean gene expression level of PD patients resembled similar trends (up/downregulation) within the set of genes of interest, as in KO cells. This synergistic effect was the case for the cell cycle regulator *CDKN1C* (upregulated in all KO cells and IPD data sets), and genes related to the immune system such as *TLR6* (upregulated in HCT116-*PER2*^KO^ and IPD data sets), *SERPINA1* (upregulated in all KO cells and the IPD data sets) and the PD-associated gene *GBA* (involved in energy metabolism), synergistically upregulated in IPD data sets and HCT116-*ARNTL*^KO^, HCT116-*NR1D1*^KO^. *TGFB1* involved in cell proliferation, inflammation and differentiation exhibited a synergistic effect in HCT116-*PER2*^KO^ and HCT116-*NR1D1*^KO^, but an antagonistic effect in HCT116-*ARNTL*^KO^, as compared to the IPD data sets. This points to a core-clock-specific effect on gene expression changes and highlights the fact that the regulation of these differentially expressed genes (common between HCT116 cells and IPD samples) might affect both diseases (cancer and neurodegeneration) in a different manner.

Another interesting example is the gene *SNCA*, which encodes for the protein α-synuclein. This protein was found to be accumulated in patients with PD and linked to Lewy bodies. Mutations in *SNCA* have been reported in PD patients with a genetic background [148] and elevated levels of *SNCA* were found in IPD [149] and shown to vary during the course of disease progression [150]. Moreover, *SNCA* mutations are responsible for a rare autosomal dominant form of PD that shows severe fluctuations and prominent psychiatric features including hallucinations and autonomic dysfunction [147]. Our results showed upregulation of *SNCA* only in *ARNTL*^KO^ cells, whereas it was downregulated in *NR1D1*^KO^ cells, as well as in IPD patients. This suggests that perturbation of core-clock genes may result in differential outputs in *SNCA* expression levels. The specific clinical profile of patients with either *GBA* or *SNCA* mutations with prominent motor and nonmotor fluctuations and the results from our analysis provides a putative link between circadian dysfunction and these important clinical symptoms.

Our results further point to sex-specific variations in gene expression within the PD cohort. Female patients showed more genes to have expression changes in the same direction as in the clock KO cells, as compared to male patients. *DBP*, a clock gene, was differentially expressed uniquely in females. Its rhythmic pattern was abolished in *ARNTL*^KO^ cells and upregulated in *PER2*^KO^ and *NR1D1*^KO^ cells. Previous studies have shown the loss of circadian expression of *Dbp* in *Arntl* KO mice, and manipulation of *Arntl* was found to be tightly linked to neurodegeneration [151]. Furthermore, a study investigating changes in colonic mucosa of healthy and neoplasmic mice suggested reduction in amplitudes and phase delay in circadian expression of *DBP* in colonic mucosa of healthy mice compared to colonic neoplasm [152], thus strengthening the potential role of the circadian clock in the crosstalk of the two pathologies. Even though no additional clock gene was differentially expressed in the cohort of IPD patients, our data showed a weaker correlation of CCN and ECCN genes in IPD when compared to controls (sex separated), pointing to a change in the relative expression of clock genes due to PD. The expression of clock genes has been shown to be altered in PD, as already discussed [22,48,50]. The overall lack of available time series data in the cohort of IPD patients and the lack of information regarding the timing of blood sampling in the IPD dataset analyzed did not allow us to carry out a circadian analysis of the data sets. Nevertheless, to overcome this limitation, we have performed differential expression analysis using both the CRC and IPD data sets, and we have additionally analysed circadian profiles of our genes of interest among the top 100 differentially IPD expressed genes in our CRC data. Out of these genes, we observed a gain of oscillations in *DAPK2* (in *ARNTL*^KO^ cells), *ATP6V0D1* (in *NR1D1*^KO^ cells) and *CXCL16* (in *PER2*^KO^ cells) compared to HCT116^WT^ cells, whereas a loss of rhythmicity was observed for *GBA* and *CYBA* for all KO conditions. Taken together, our study shows that IPD patients and core-clock KO CRC cells resemble similar trends in alteration of gene expression for the pre-defined genes of interest and suggest a role for the circadian clock in the regulation of molecular pathways that are altered in cancer and neurodegeneration. However, the generation of time-course data sets for PD patients in future studies is needed and may allow for a better identification of molecular signatures for prognostic and diagnostic purposes in PD, by also considering the circadian profile of disease-associated genes.

Our analysis pointed to a link between the genes that resulted from the time-series analysis in CRC cells and the differential expressed genes in the IPD datasets, and cancer hallmarks genes retrieved from CHG database [56]. Namely, we found associations to “resistance to cell death”, “sustaining proliferative signaling” and “activation of invasion and metastasis”, which reinforces our initial hypothesis of a correlation regarding common altered pathways both in cancer and neurodegeneration.

Furthermore, a subsequent analysis on the possible role of the altered genes in CRC and IPD data sets in the survival of cancer patients (TCGA-COAD data sets) pointed to a significant influence on survival for eight of these genes (*TUBB6*, *PAK6*, *SULT1A1*, *SLC11A1*, *TRAP1*, *FAM50B*, *SPON2*, *FES*). Our results showed that downregulation of *PAK6* resulted in a higher overall survival probability in colon cancer patients. In line with our observation, *PAK6* was reported to be associated with overall survival, enhancing chemotherapeutic resistance and was proposed as a prognostic marker for colon cancer patients [153]. Furthermore, it was shown that *PAK6* interacts with *LRRK2* whose mutation is linked to a genetic form of PD and its aberrant activation was observed in IPD [154,155,156]. Interestingly, *PAK6* was upregulated in *ARNTL*^KO^, *NR1D1*^KO^ cells and IPD female patients, suggesting a potential function for *PAK6* linking cancer and neurodegeneration. Another significant example is *TUBB6* whose downregulation yielded a better survival probability in TCGA-COAD cohort and was found to lose rhythmicity in all KOs, in our analysis. *TUBB6* was previously proposed as a prognostic biomarker in several cancer types including gastric [157], ovarian [158] and prostate cancer [159]. Furthermore, *TUBB6* was found to be associated with inflammatory induced cell death response [160] and reported to be also a direct interactor with *LRRK2* kinase [161], which is involved in PD [144,156], as described above. The loss of rhythmic expression of *TUBB6* points to a regulation of this gene via the circadian clock. These findings suggest that several of the genes highlighted in our study are likely relevant in a colorectal cancer and IPD context, impact the survival of cancer patients and highlight potential novel links between cancer and neurodegeneration. Future experiments will be needed to further validate and investigate the exact role of circadian regulation (or the lack of it) of these genes in the context of different cancer types and PD. It is relevant to point out that even though we have carried out a survival analysis by also accounting for tumour stage, which highlighted this trait as a relevant factor contributing to survival, additional clinical traits, such as treatment regime, may also contribute to the prognostic potential of these candidates.

## 5. Conclusions

Empirical evidence of circadian dysfunction in PD is only beginning to be unveiled. Many studies from human subjects show conflicting results or rely on findings that are intrinsically multifactorial in origin. Further research requires the generation of time course data sets for PD patients and should focus on describing in greater detail the mechanisms underlying circadian dysfunction, its association and causative role in many of the clinical and physiological changes detected in these patients. Care should be taken when evaluating the circadian system in PD patients due to the possible reciprocal influence of neuropathophysiological variability and the circadian system. It is also important to point out that considering patient stratification based on specific clinical traits, prior to the analysis, may influence the results presented in this study, as shown by our analysis regarding the significance of factors like age, sex, and stage. A potentially relevant, but mostly neglected, clinical trait is the patients’ circadian rhythm, which should be considered in future studies, and for which new methods for its accurate assessment and monitoring need to be further developed.

## Figures and Tables

**Table 1:** Clinical characterization of the IPD patients. MoCA; NA = Not Applicable. The clinical characteristics listed in Table 1 correspond to the entire dataset downloaded (GEO: GSE99039). For the subsequent analysis, only 204 PD patients and 227 controls were retained (see Materials and Methods, Section 2.4 for further details).

Sample Size	205 IPD Patients	233 Controls
Sex	101 males; 90 females; 14 samples without sex information	70 males; 142 females; 21 samples without sex information
Age (all patients)	62 ± 11 (mean ± SD)	58 ± 30 (mean ± SD)
Age at disease onset (all patients)	56 ± 11 (mean ± SD)	NA
MoCA	27 ± 3	NA
Hoehn and Yahr stage		
0	7	NA
1	58	NA
2	70	NA
3	30	NA
4	8	NA
5	0	NA
Missing data	32	NA

## Data Availability

RNA-sequencing data (HCT116 WT and KO cell lines) was submitted to the ArrayExpress repository (E-MTAB-9701) and will be released upon publication of the manuscript.

## Supplementary Materials

The following are available online at https://www.mdpi.com/article/10.3390/cancers13235978/s1. Figure S1: Heatmap for ECCN genes in HCT116 cells, Figure S2: Biological processes associated with 12-h rhythmic genes in HCT116 cells, Figure S3: Circadian expression profiles of genes of interest in SW480 and SW620 cells, Figure S4: Biological processes associated with differentially expressed genes in HCT116 cells, Figure S5: Biological processes associated with differentially expressed genes in IPD datasets, Figure S6: Heatmap for differentially expressed genes of interest in HCT116 cells, Figure S7: Commonly differentially expressed genes of interest in HCT116 KOs and males in IPD cohort, Figure S8: Commonly differentially expressed genes of interest in HCT116 KOs and in female and male IPD patients, Figure S9: Heatmap of top 100 differentially expressed genes (sorted by p-value) in males, Figure S10: Expression patterns of ECCN genes in IPD female patients, Figure S11: Expression patterns of ECCN genes in IPD patients, Figure S12: Expression patterns of ECCN genes in IPD male patients, Figure S13: Circadian expression profiles of genes of interest among the top 100 differentially expressed genes in the IPD cohort (without sex separation, top panel), in females (middle panel) and in males (bottom panel), Figure S14: Significance analysis on the impact of clinical traits potentially relevant for patient survival, Figure S15: Survival curves for short-listed genes associated with colon cancer patient survival, Table S1: Demographic information of GSE99039 dataset downloaded from GEO (date of data retrieval: 16.02.2021) and ArrayQualityMetrics outlier test results (failed tests are marked with “x”), Table S2: List of genes pre-selected from KEGG pathways in immune system module, Table S3: List of genes pre-selected from KEGG pathways DNA replication and repair, Table S4: List of genes pre-selected from KEGG pathways involved in cellular growth, Table S5: List of genes pre-selected from KEGG pathways involved in redox homeostasis (energy metabolism and catabolism), Table S6: List of genes from the ECCN, Table S7: List of genes pre-selected from KEGG pathways involved in PD and genes involved in PD-associated disorders restless legs syndrome (RLS) with possible circadian influences, Table S8: GO Biological Process Enrichment results for 24-h and 12-h rhythmic genes, Table S9: GO Biological Process Enrichment results for differentially expressed genes in HCT116 cells, Table S10: GO Biological Process Enrichment results for differentially expressed genes in the IPD Dataset, Table S11: List of genes from the analysis with CRC and IPD data sets, Table S12: The list of differentially expressed genes found to be associated with overall survival of TCGA-COAD patients and results from the Cox Regression analysis based on stratification for tumour stage.
