# Supplementary material for: A Computational Analysis in a Cohort of Parkinson’s Disease Patients and Clock-Modified Colorectal Cancer Cells Reveals Common Expression Alterations in Clock-Regulated Genes
Source: Cancers (Basel). 2021 Nov 28;13(23):5978. doi: 10.3390/cancers13235978 (PMC8657387; doi:10.3390/cancers13235978)
Supplement: Supplementary file 1 [file cancers-13-05978-s001.zip › Supplementary_Files/Yalcinetal_2021_Cancers_Supplementary_File.pdf]

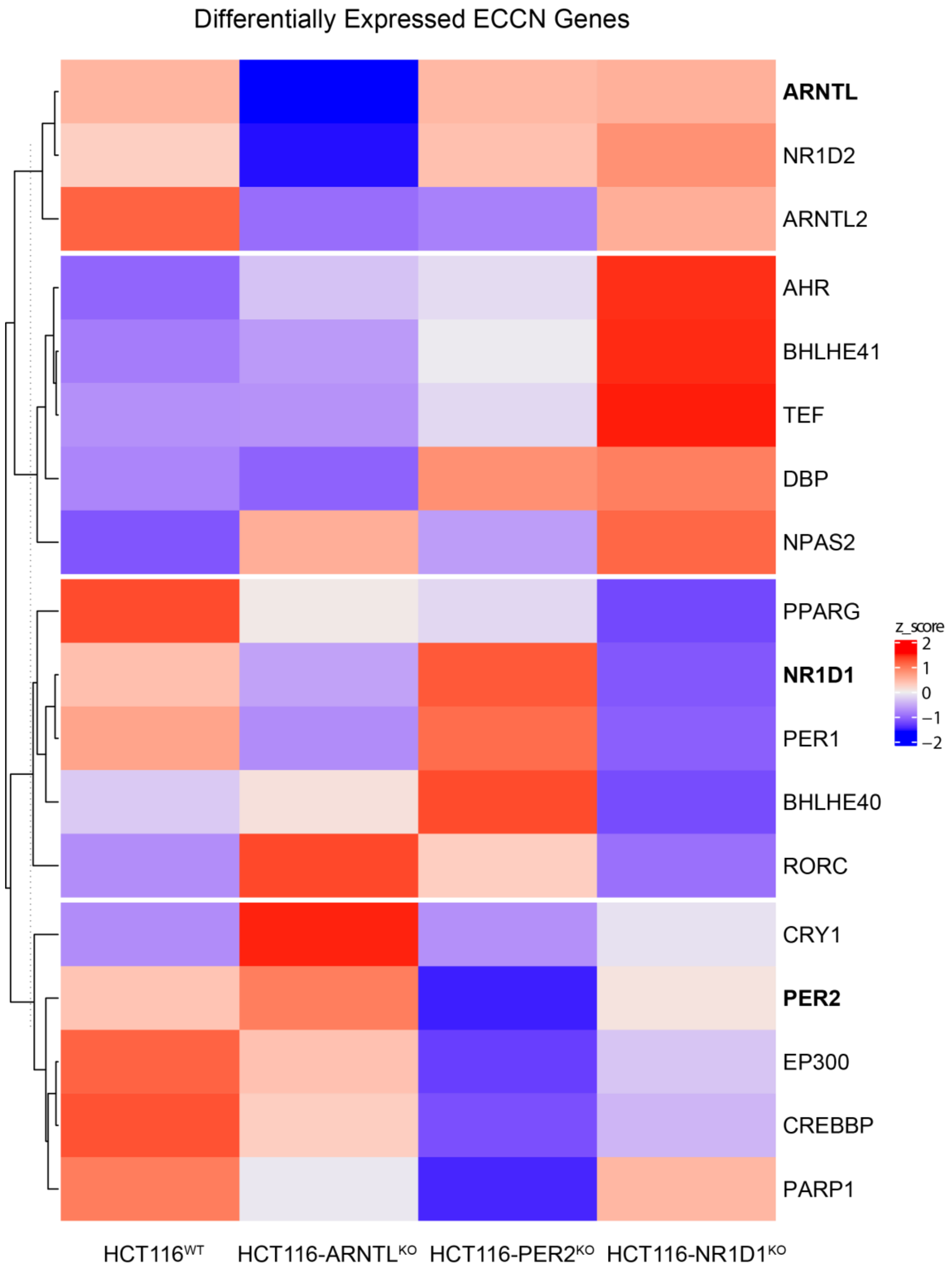

Figure S1. Heatmap of ECCN genes in HCT116 cells. *ECCN genes that were differentially expressed in at least one of the Knock Out (KO) cells compared to HCT116WT cells are depicted in the heatmap. KO genes (ARNTL, PER2 and NR1D1) are depicted in bold. Clustering was performed using k-means clustering (k=4). Colour code scheme for standardized gene expression values (z-scores) are indicated in the heatmap colour key.*

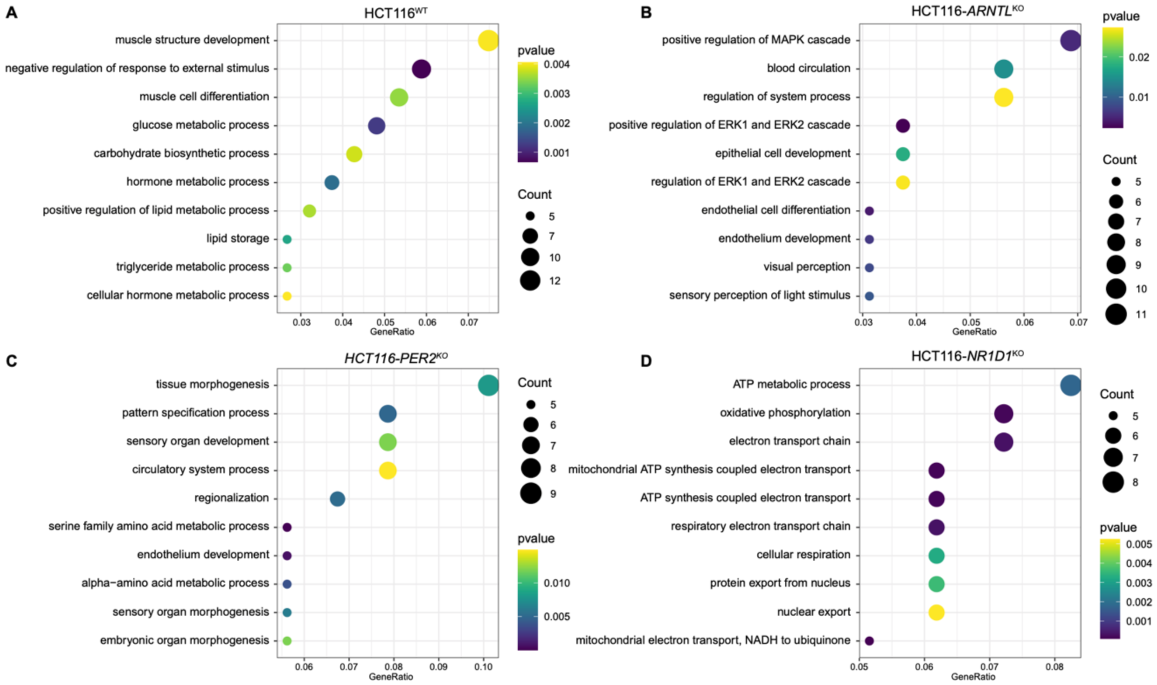

Figure S2. Biological processes associated with 12h rhythmic genes in HCT116 cells. *Top 10 GO (Biological Processes) enriched in **(A)** HCT116^{WT} cells, **(B)** HCT116-ARNTL^{KO} cells **(C)** HCT116-PER2^{KO}cells **(D)** HCT116-NR1D1^{KO} cells.*

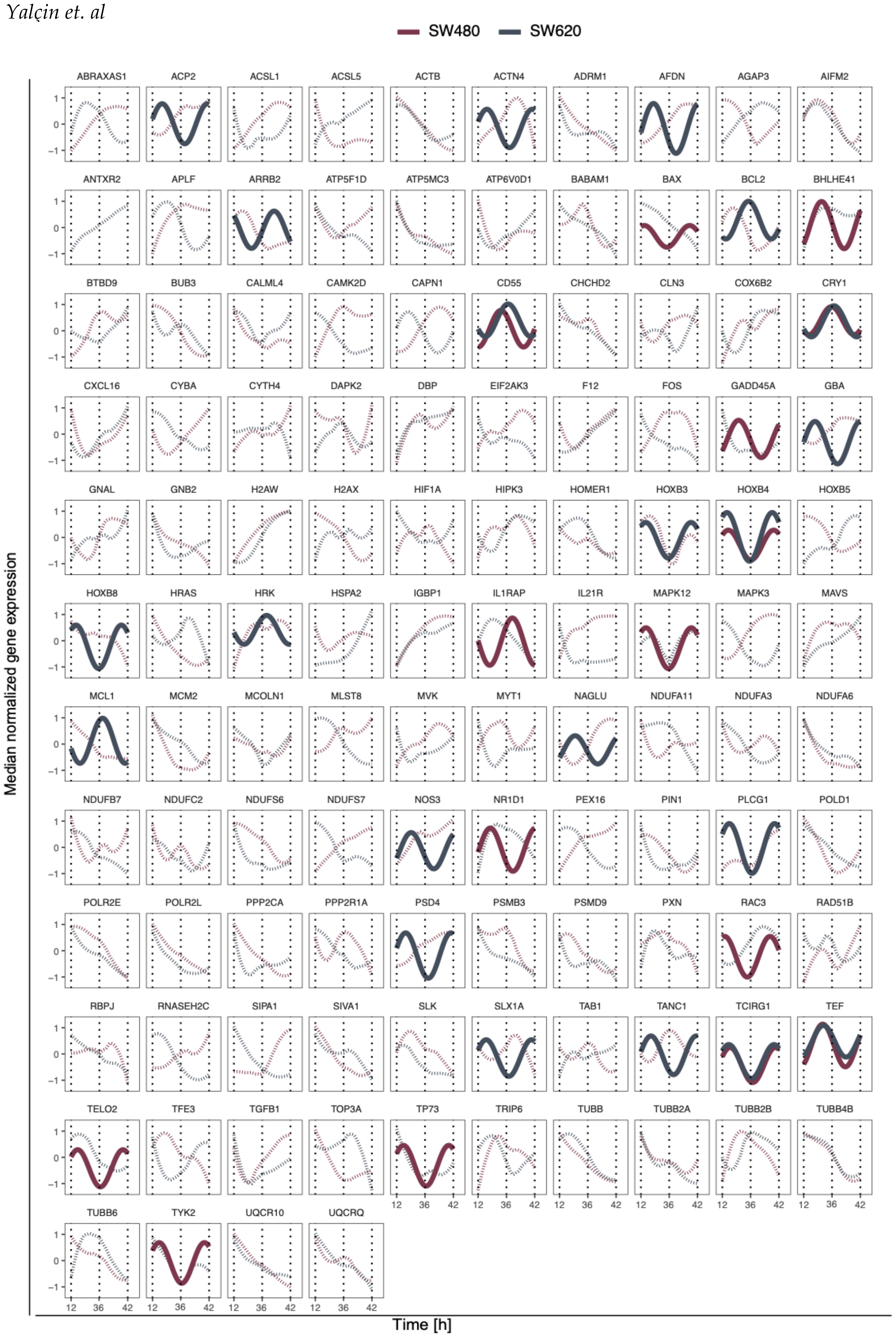

Figure S3*. *Circadian expression profiles of genes of interest in SW480 and SW620 cells. *Rhythmicity analysis was computed considering 24h-period. Genes that were found to be significantly rhythmic (q<0.05, relative amplitude ≥ 0.1) plotted using a harmonic regression curve (full lines) and non-significant ones are shown with loess smoothened lines (dashed lines).*

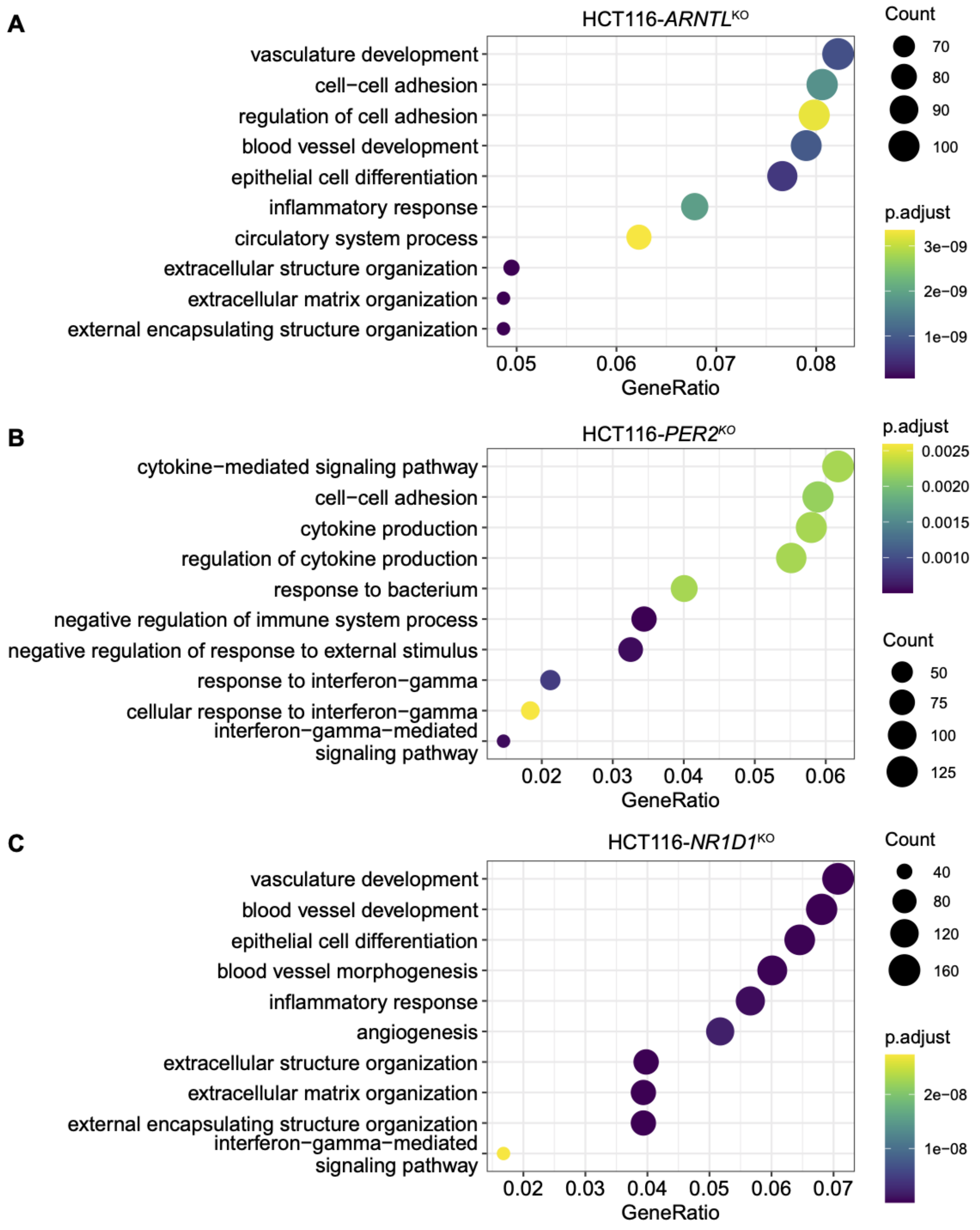

Figure S4. Biological processes associated with differentially expressed genes in HCT116 cells. *Top 10 GO (Biological Processes) enriched in* ***(A)*** *HCT116-ARNTL^{KO} cells vs HCT116^{WT} cells* ***(B)*** *HCT116-PER2^{KO} cells vs HCT116^{WT} cells* ***(C)*** *HCT116-NR1D1^{KO} cells vs HCT116^{WT} cells.*

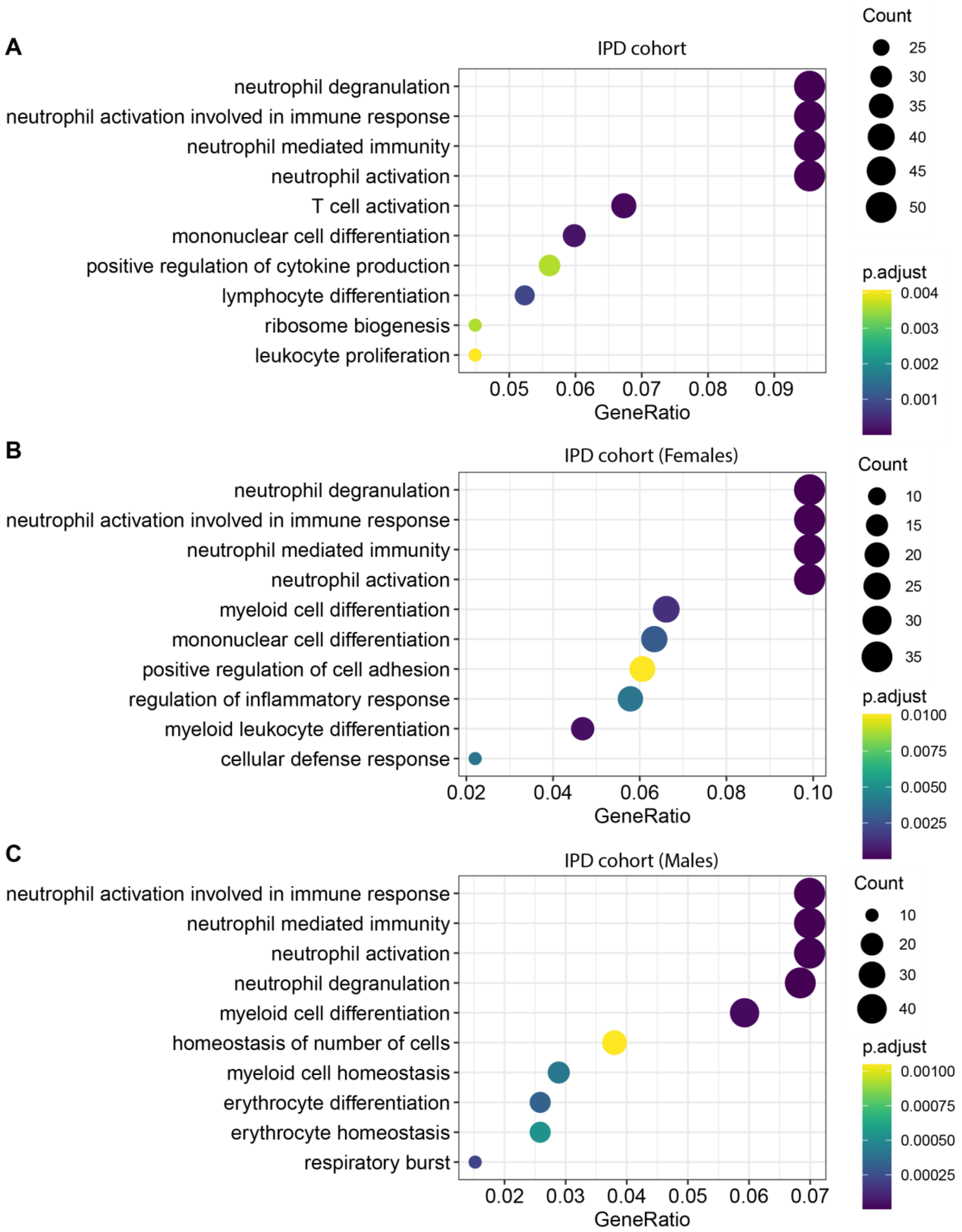

Figure S5. Biological processes associated with differentially expressed genes in Idiopathic Parkinson's Disease datasets. *Top 10 GO (Biological Processes) enriched in* ***(A)*** *IPD patients vs respective controls,* ***(B)*** *Female IPD patients vs respective controls,* ***(C)*** *Male IPD patients vs respective controls.*

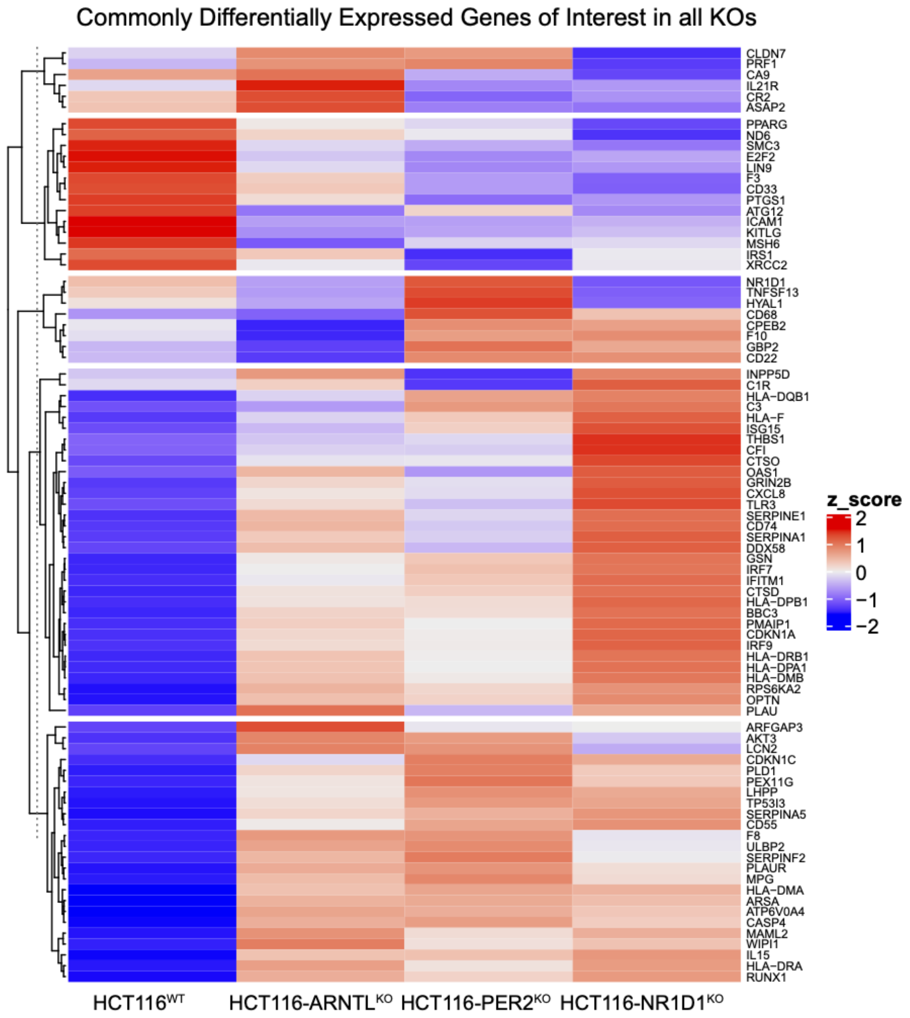

Figure S6. Heatmap of differentially expressed genes of interest in HCT116 cells. *Genes of interest that were differentially expressed in all Knock Out (KO) cells compared to HCT116^{WT} cells are depicted in the heatmap. Clustering was performed using k-means clustering (k=4). Colour code scheme for standardized gene expression values (z-scores) are indicated in the heatmap colour key.*

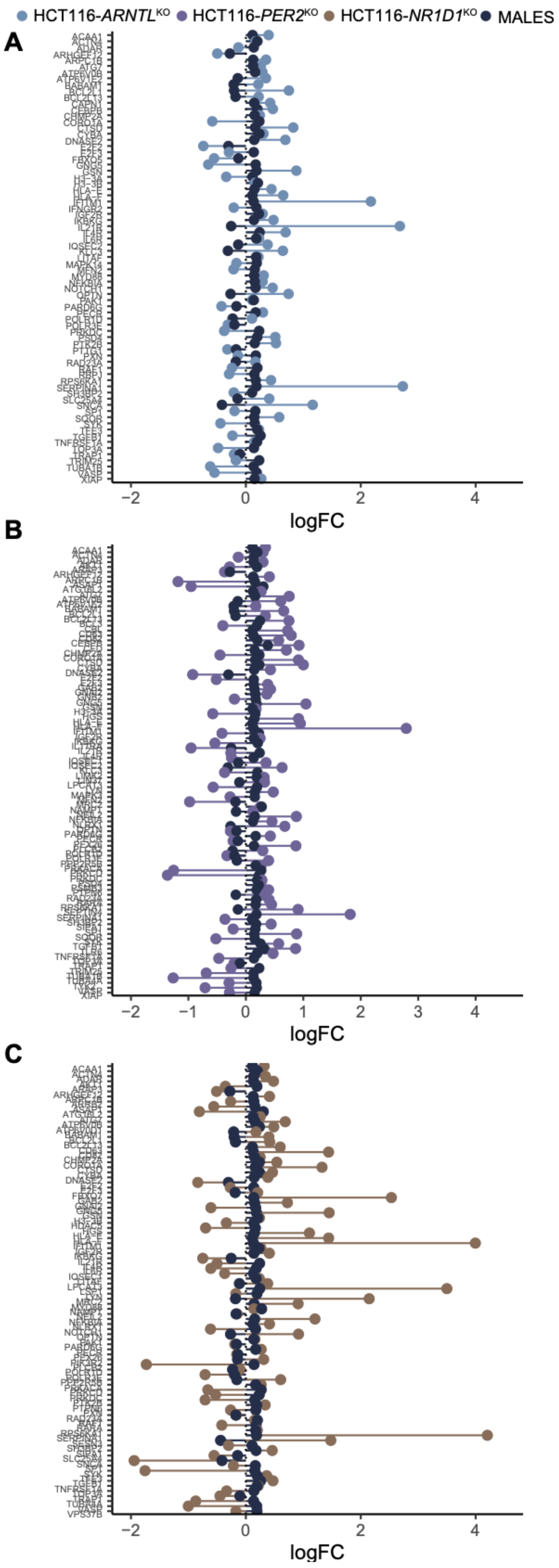

Figure S7. Commonly differentially expressed genes of interest in HCT116 KOs and males in IPD cohort. *Lollipop plots showing the direction of change in mean expression level of genes of interest in **(A)** HCT116-ARNTL^{KO} cells vs HCT116^{WT} cells and male IPD patients vs controls **(B)** HCT116-PER2^{KO} cells vs HCT116^{WT} cells and male IPD patients vs controls **(C)** HCT116-NR1D1^{KO} cells vs HCT116^{WT} cells and males IPD patients vs controls.*

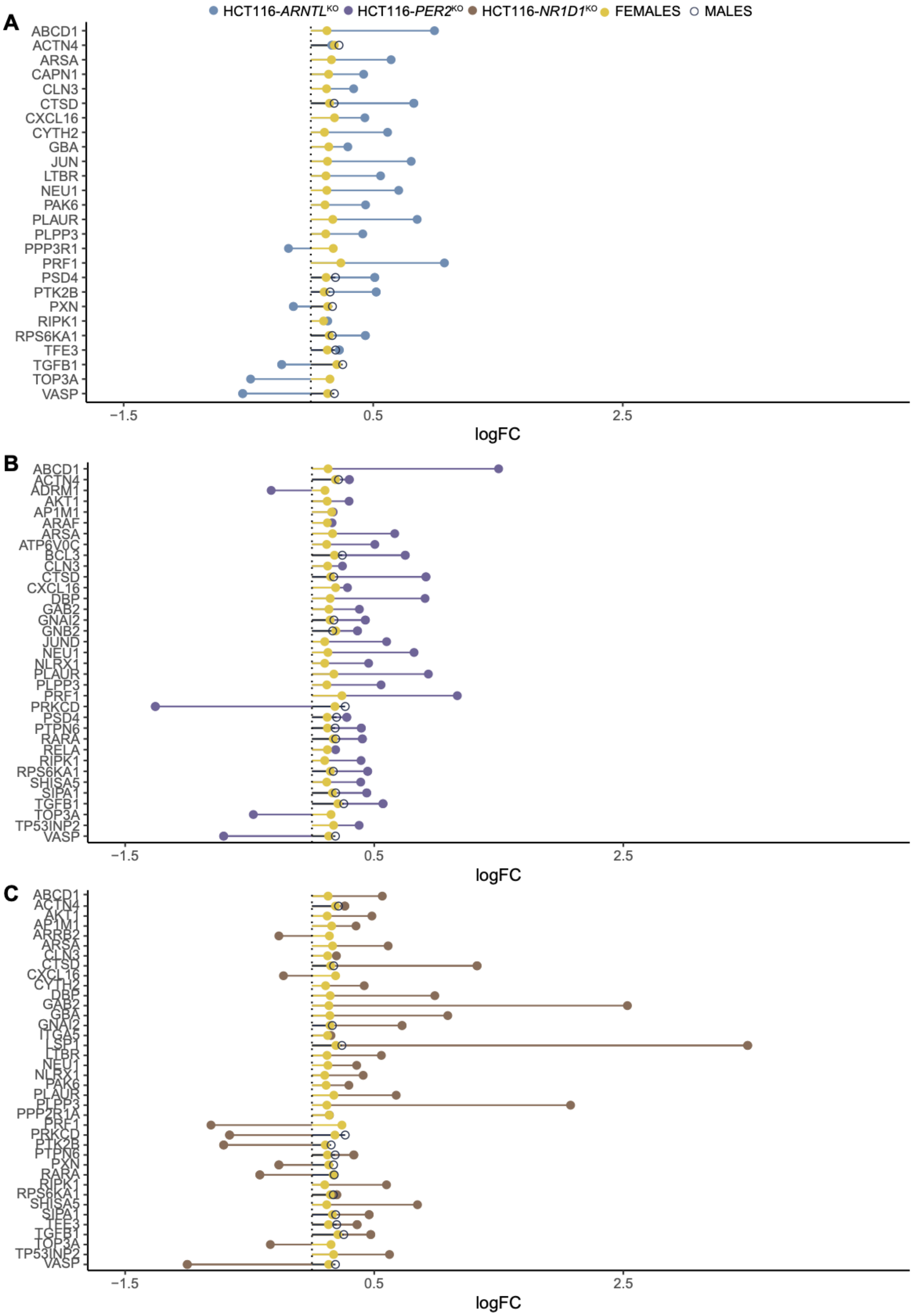

Figure S8. Commonly differentially expressed genes of interest in HCT116 KOs and in females and male cohort. *Lollipop plots showing the direction of change in mean expression level of genes of interest that were found in the intersection between females and males in IPD cohort vs controls and* ***(A)*** *HCT116-ARNTL^{KO} cells vs HCT116^{WT} cells* ***(B)*** *HCT116-PER2^{KO} cells vs HCT116^{WT} cells* ***(C)*** *HCT116-NR1D1^{KO} cells vs HCT116^{WT} cells .*

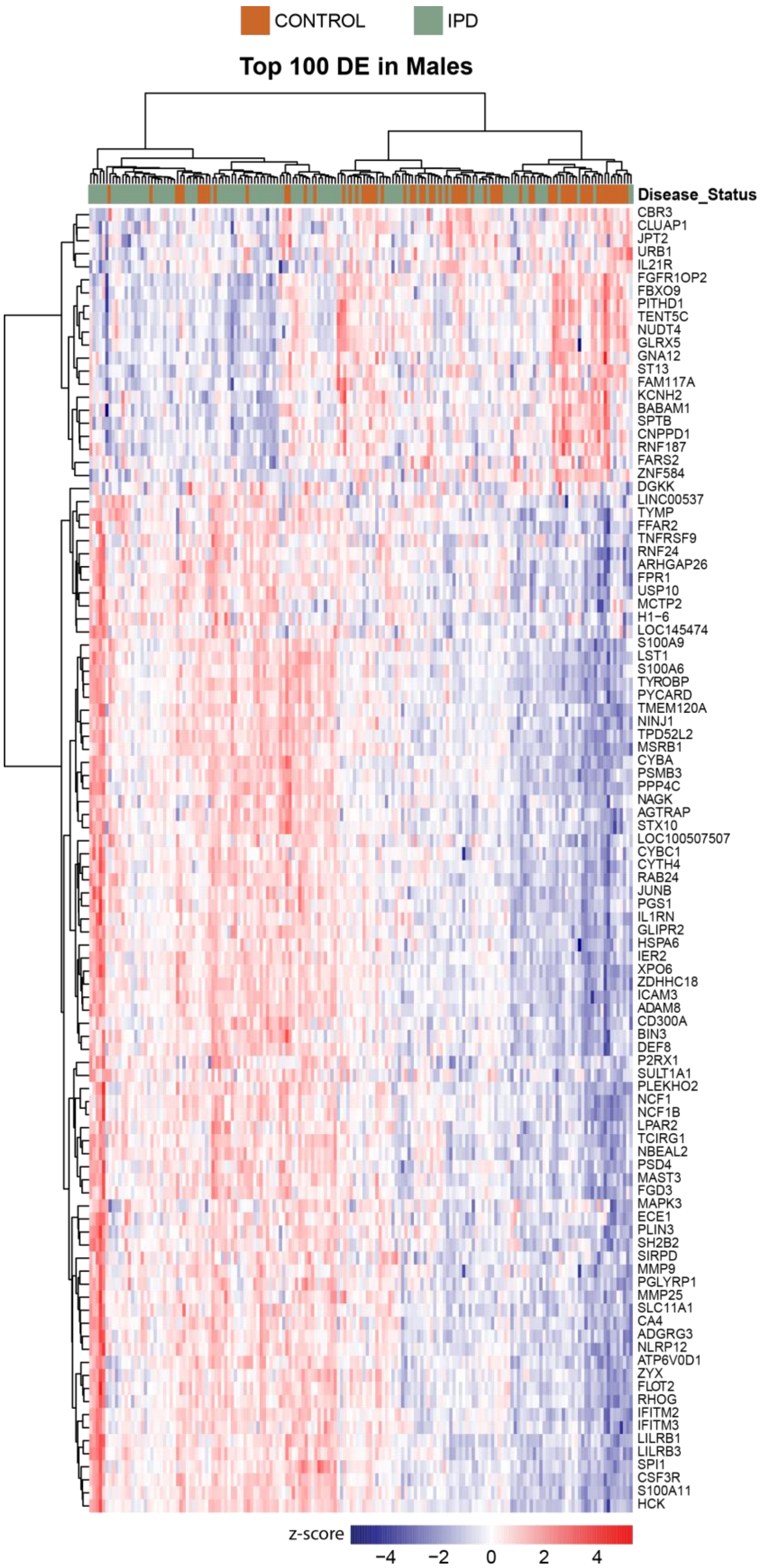

Figure S9. Heatmap of top 100 differentially expressed genes (sorted by p-value) in males. *The heatmap was clustered using WardD2 linkage and Euclidean distancing. Disease status of the samples denoted as orange for controls and green for patients. Colour code scheme for standardized gene expression values (z-scores) are indicated in the heatmap colour key.*

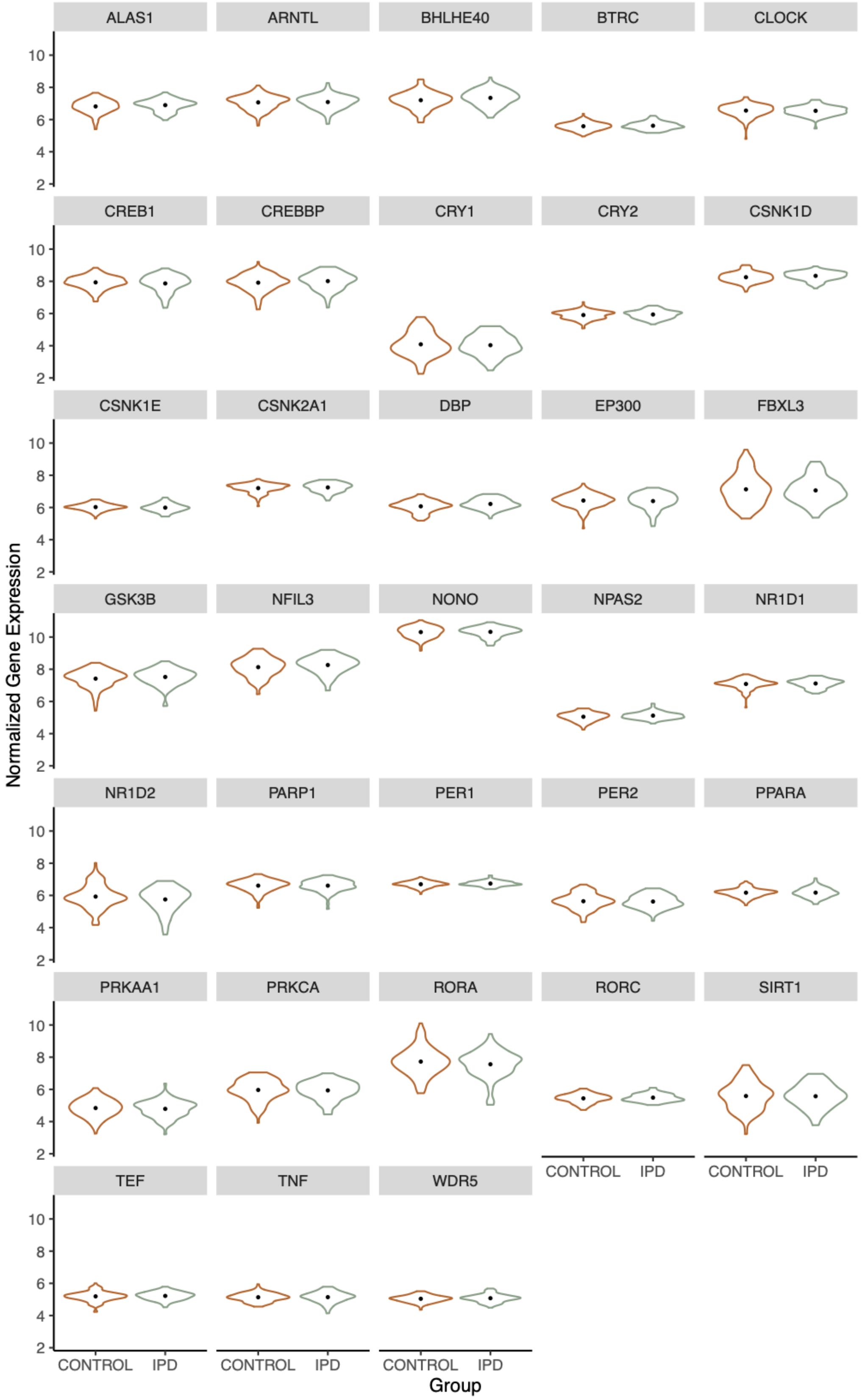

Figure S10. Expression patterns of ECCN genes in female cohort. *Violin plots used to depict the distribution of individual gene expression levels of ECCN genes in females (N_{IPD}=90, $N_{Controls}$=138). Mean gene expression of total population is depicted with black dots. The disease status is denoted as orange for controls and green for patients.*

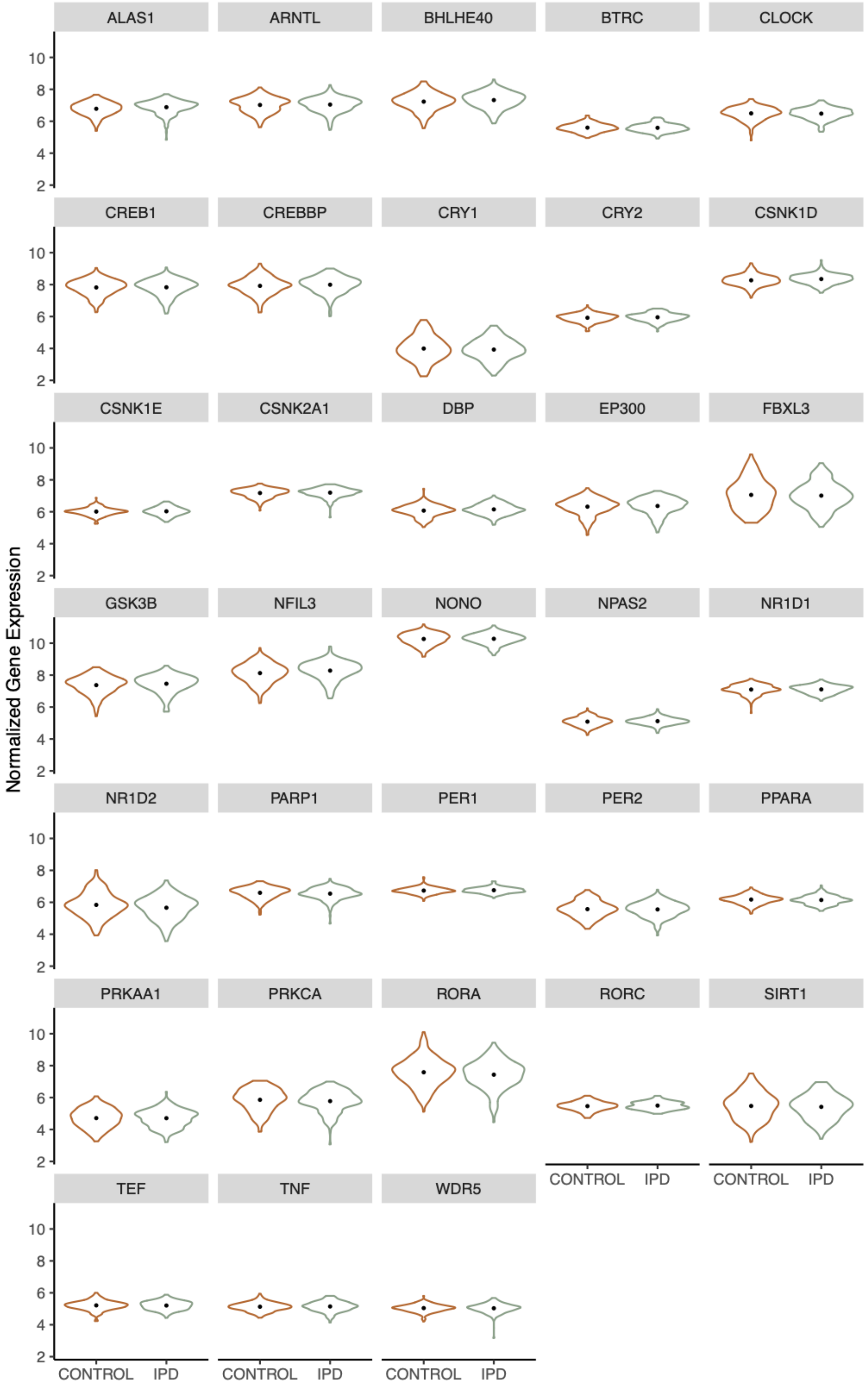

Figure S11. Expression patterns of ECCN genes in IPD cohort. *Violin plots used to depict the distribution of individual gene expression levels of ECCN genes in patient cohort ($N_{Controls}$=227, N_{IPD}=204). Mean gene expression of total population is depicted with black dots. The disease status is denoted as orange for controls and green for patients.*

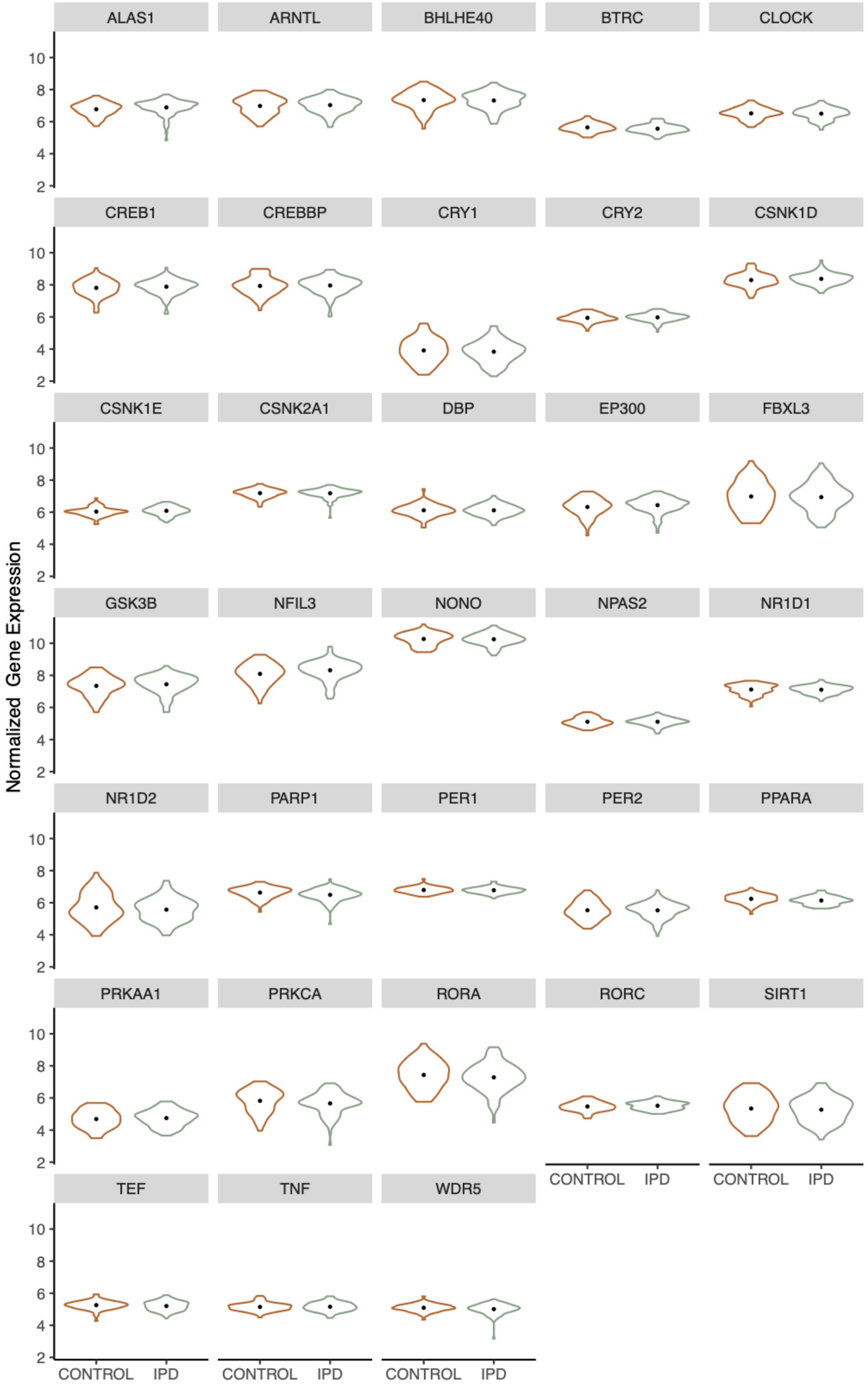

Figure S12. Expression patterns of ECCN genes in male cohort. *Violin plots used to depict the distribution of individual gene expression levels of ECCN genes in males (N_{IPD}=100, $N_{Controls}$=69). Mean gene expression of total population is depicted with black dots. The disease status is denoted as orange for controls and green for patients.*

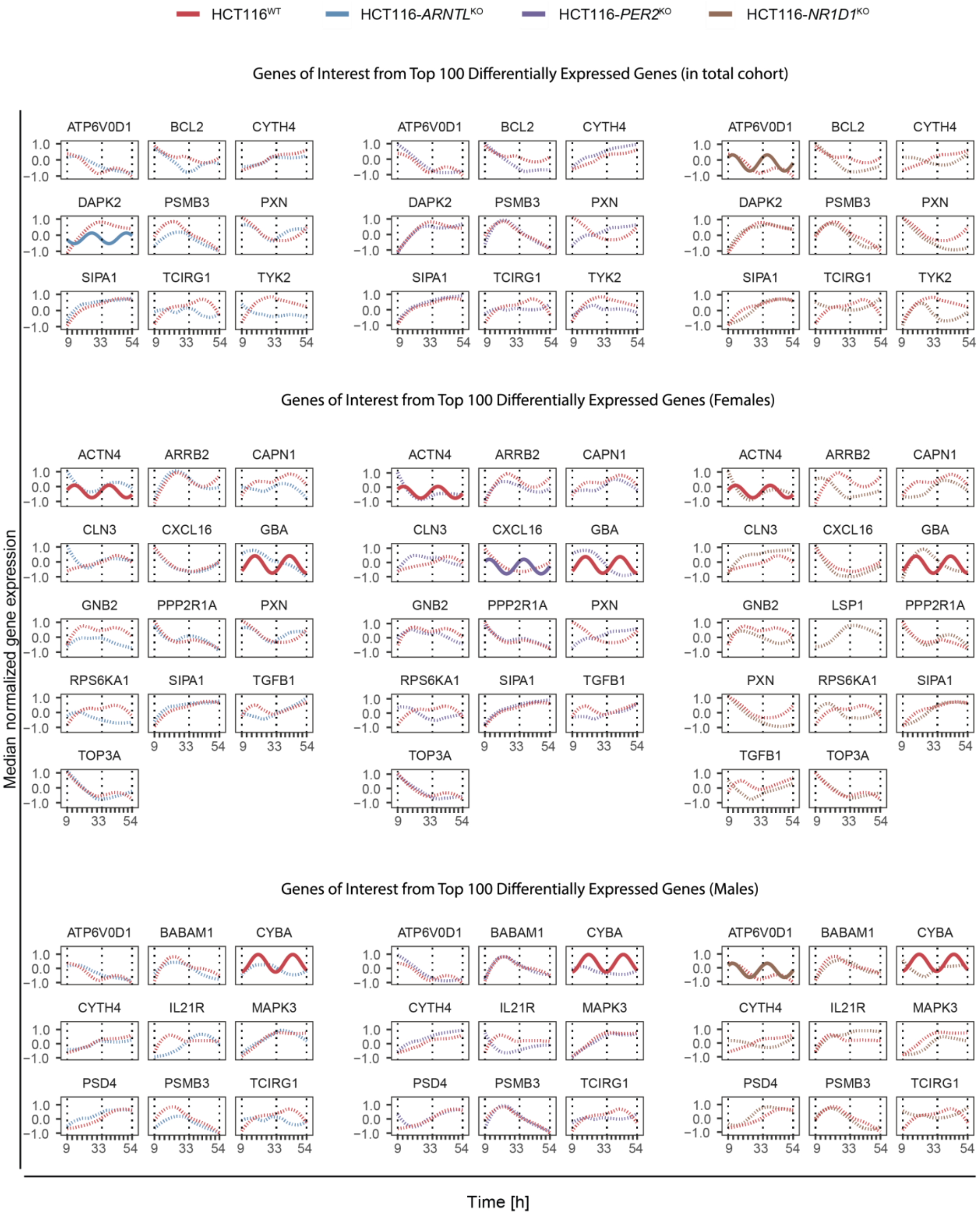

Figure S13. **Circadian expression profiles of genes of interest among the top 100 differentially expressed genes in the IPD cohort (without sex separation, top panel), in females (middle panel) and in males (bottom panel).** Rhythmicity analysis was computed considering 24h-period. Depicted are significantly rhythmic genes ($q < 0.05$ and relative amplitude ≥ 0.1) plotted using a harmonic regression fitting (full lines) whereas non-significantly rhythmic genes were represented with LOESS (dashed lines) (see **Figure S2** for similar analysis in SW480 and SW620 cells).

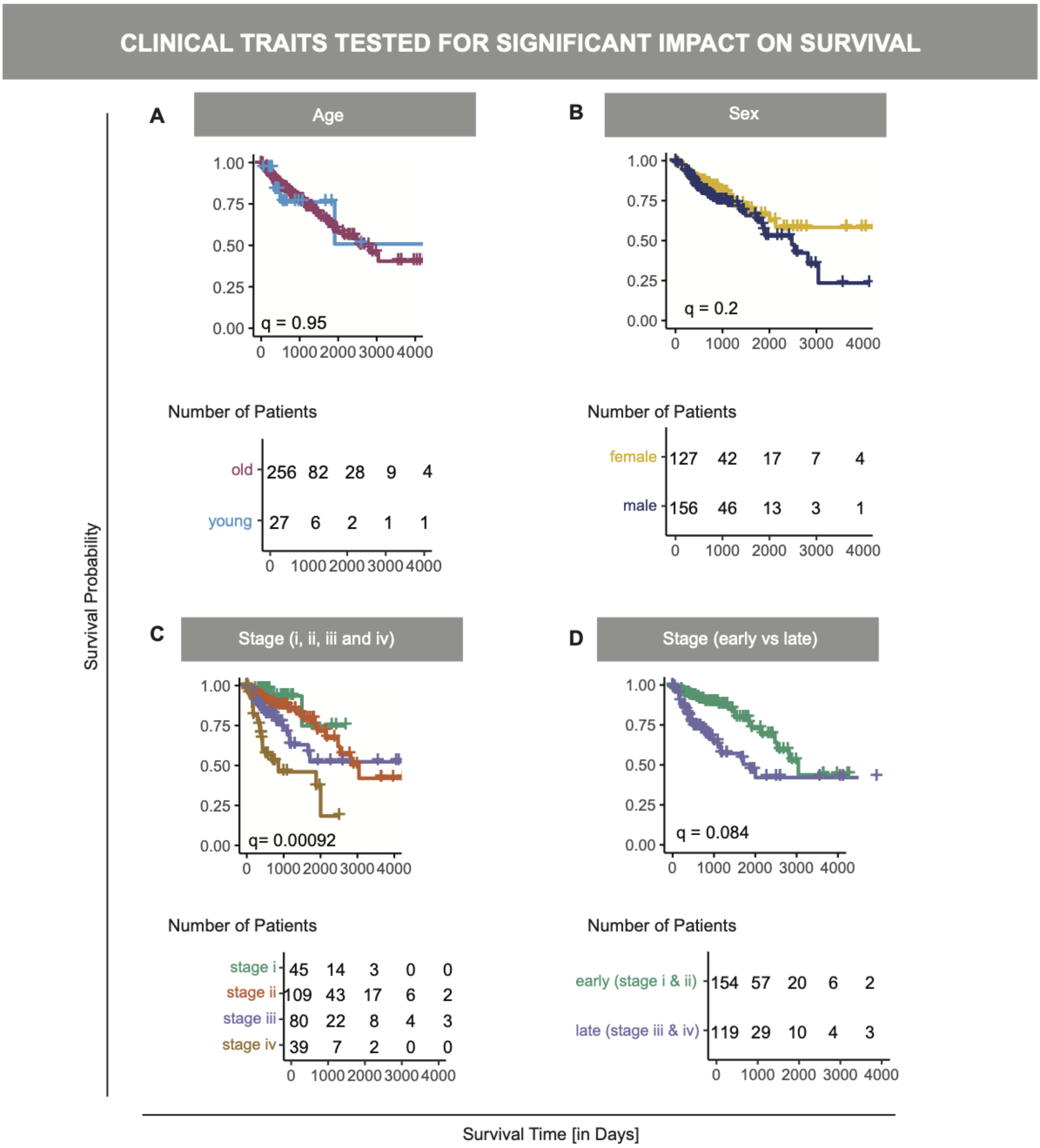

Figure S14. Significance analysis on the impact of clinical traits potentially relevant for patient survival. *Kaplan Meier curves represent patient survival in subgroups of the TCGA COAD cohort based on different clinical trait including* ***(A)*** *age ;* ***(B)*** *sex ;* ***(C)*** *disease stage (I,ii,iii,iv) and* ***(D)*** *early stage (I and ii) versus late stage (stage iii and iv).The tables show number of patients (population sizes) in each sub-group for different survival intervals (in days).*

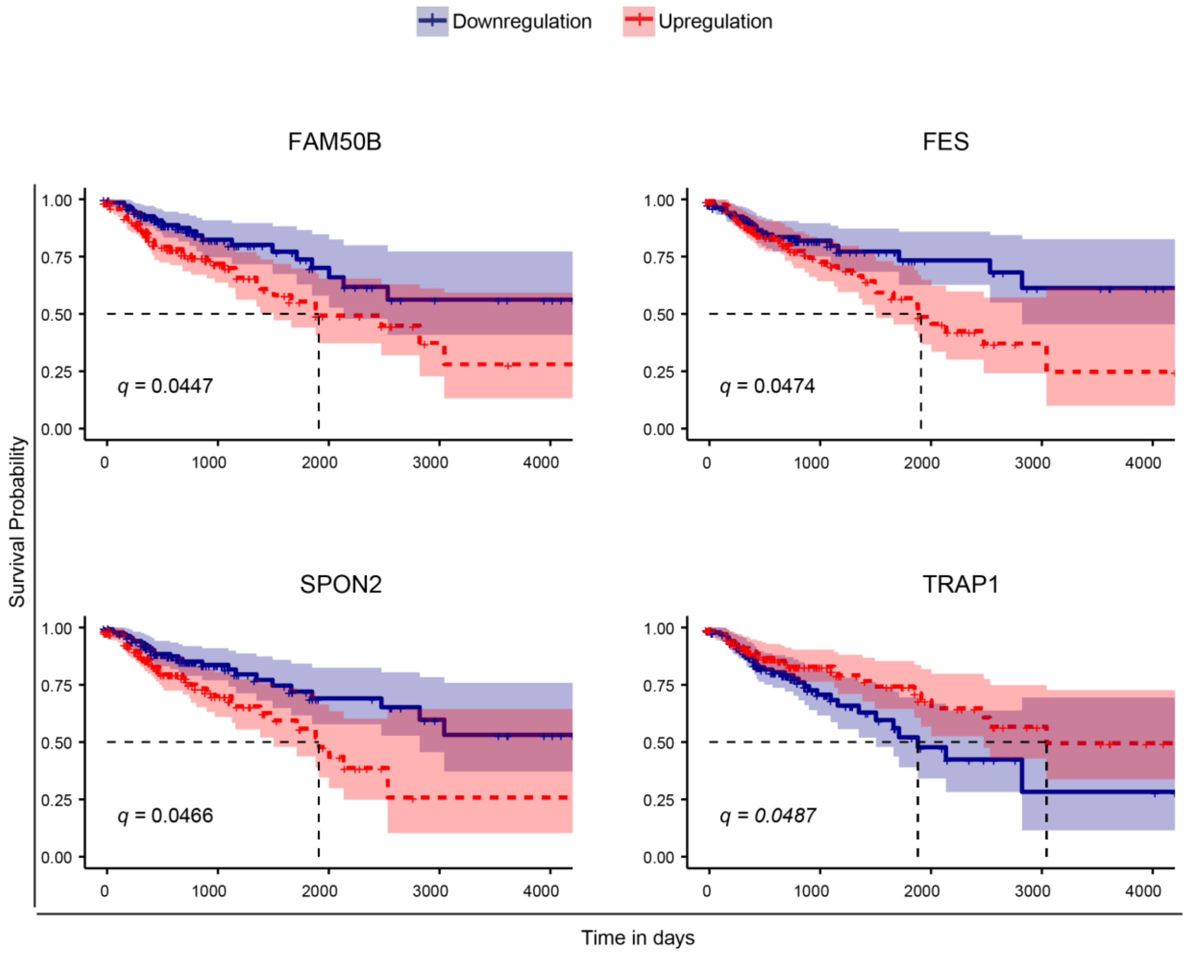

Figure S15. Survival curves of short-listed genes associated with patient survival. *Kaplan Meier survival curves for genes found to be common in our analysis and genes associated with patient survival in TCGA COAD cohort. The patient cohort was separated into two groups based on the mean gene expression level as high and low expression group and a subsequent comparison of survival time (in days) was performed. Stage stratified q-values for the survival analysis with cox-regression yielded the results: FAM50B : q = 0.312 ; FES : q = 0.1 ; SPON2 : q = 0.143 ; TRAP1 : q= 0.042*

Supplementary Table 1. *Demographic information of GSE99039 dataset downloaded from GEO (the date of data retrieval 16.02.2021) and ArrayQualityMetrics outlier test results (failed tests are marked with "x")*

Supplementary Table 2. *The list of genes pre-selected from KEGG pathways in immune system module*

Supplementary Table 3. *The list of genes pre-selected from KEGG pathways DNA replication and repair*

Supplementary Table 4. *The list of genes pre-selected from KEGG pathways involved in cellular growth*

Supplementary Table 5. *The list of genes pre-selected from KEGG pathways involved in redox homeostasis (energy metabolism and catabolism)*

Supplementary Table 6. *The list of Extended Core-Clock Network*

Supplementary Table 7. *The list of genes pre-elected from KEGG pathways involved in Parkinson's Disease and genes involved in PD associated disorders Restless legs syndrome (RLS) with possible circadian influences*

Supplementary Table 8. *GO Biological Process Enrichment results of 24h and 12h rhythmic genes*

Supplementary Table 9. *GO Biological Process Enrichment results of differentially expressed genes in HCT116 cells*

Supplementary Table 10. *GO Biological Process Enrichment results of differentially expressed genes in Parkinson's Disease Dataset*

Supplementary Table 11. *The list of genes resulted from the analysis with CRC and IPD data sets*

Supplementary Table 12. *The list of differentially expressed genes found to be associated with overall survival of TCGA-COAD patients and results from the cox-regression analysis based on stratification for tumor stage*
